# Spanish consensus on the diagnosis and management of adrenocortical carcinoma

**DOI:** 10.1530/ERC-25-0034

**Published:** 2025-04-24

**Authors:** Marta Araujo-Castro, Cristina Álvarez-Escola, Ana Casteràs, Alberto Carmona-Bayonas, María-Dolores Chiara, Felicia A Hanzu, Jorge Hernando, José L Vercher-Conejero, Macarena Rodríguez-Fraile, Victoria Gómez Dos Santos, Paula Jimenez-Fonseca, Alexandra Giraldo, Nuria Valdés, Oscar Vidal, Maribel Del Olmo-García, Jaume Capdevila

**Affiliations:** ^1^ Endocrinology & Nutrition Department. Hospital Universitario Ramón y Cajal Madrid, Madrid, Spain; ^2^ Instituto de Investigación Biomédica Ramón y Cajal (IRYCIS), Madrid, Spain; ^3^ Endocrinology & Nutrition Department. Hospital Universitario La Paz, Madrid, Spain; ^4^ Endocrinology & Nutrition Department. Hospital Universitario Vall d´Hebron, Barcelona, Spain; ^5^ Medical Oncology Department, Hospital General Universitario Morales Meseguer, University of Murcia, IMIB, Murcia, Spain; ^6^ Health Research Institute of the Principality of Asturias, Oviedo, Spain; ^7^ Institute of Oncology of the Principality of Asturias, University of Oviedo, Oviedo, Spain; ^8^ Endocrinology & Nutrition Department, Hospital Clinic, University of Barcelona, IDIBPAS, CIBERDEM, Barcelona, Spain; ^9^ Medical Oncology Department, Gastrointestinal and Endocrine Tumor Unit, Hospital Universitario Vall D'Hebron, Vall Hebron Institute of Oncology (VHIO), Barcelona, Spain; ^10^ Nuclear Medicine Department, Hospital Universitario de Bellvitge, IDIBELL, Barcelona, Spain; ^11^ Nuclear Medicine Department, Clínica Universidad de Navarra, Navarra, Spain; ^12^ Urology Department, Hospital Universitario Ramón y Cajal Madrid, Madrid, Spain; ^13^ Medical Oncology Department, Hospital Universitario Central de Asturias, ISPA, Oviedo, Spain; ^14^ Radiotherapy Oncology Department, Hospital Universitario Vall D' Hebron, VHIO, Barcelona, Spain; ^15^ Endocrinology & Nutrition Department, Hospital Universitario Cruces, Barakaldo, Spain; ^16^ UPV/EHU, Biobizkaia, CIBERDEM, CIBERER, Endo-ERN, Barakaldo, Spain; ^17^ General Surgery Department. Hospital Clinic de Barcelona, Barcelona, Spain; ^18^ University of Barcelona, Barcelona, Spain; ^19^ Endocrinology & Nutrition Department. Hospital Universitario y Politécnico La Fe, Valencia, Spain

**Keywords:** adrenocortical carcinoma, ENSAT, adrenalectomy, mitotane, adrenal tumor

## Abstract

Adrenocortical carcinoma (ACC) is a rare endocrine malignancy with an estimated incidence of 0.7–2 cases per million/year. The rarity of this disease, coupled with limited preclinical models and clinical trials, has hindered progress, resulting in poor outcomes, with a 5-year survival rate of approximately 35%. Currently, the only available curative treatment is complete surgical resection of the adrenal tumor. For unresectable or metastatic ACC, the current standard therapeutic modalities are mitotane, chemotherapy, radiotherapy and locoregional treatments; however, these are noncurative. Mitotane has an adrenolytic and anti-steroidogenic effect, and it is used in the adjuvant setting for high-risk patients, as systemic therapy for metastatic disease, and/or to control hormonal secretion. While key pathways in ACC pathogenesis have been identified as potential therapeutic targets, results with targeted therapies remain modest, showing that there is a clinical unmet need for novel treatments or new combinations of exiting drugs. Effective management requires a multidisciplinary team of experts to optimize outcomes for patients. This article presents a multidisciplinary consensus on the diagnosis, management, prognosis and follow-up of patients with ACC, and the approach to two special contexts, ACC in pregnant women and hormone-producing ACC. The consensus was coordinated by the Spanish Society of Endocrinology and Nutrition (SEEN) and the Spanish Group of Neuroendocrine and Endocrine Tumors (GETNE), with contribution from experts from related societies including the Spanish Association of Surgeons (AEC), Spanish Society of Urology (AEU), Anatomic-Pathology (SEAP), Nuclear Medicine (SEMNIM), Medical Oncology (SEOM) and Radiotherapeutic Oncology (SEOR).

## Introduction

Adrenal cortical tumors are common, with a prevalence of 1–10%, but most are benign adrenal adenomas discovered incidentally (Araujo-Castro *et al.* 2020). In contrast, adrenocortical carcinoma (ACC) is a rare malignancy with an incidence of 0.7–2 cases per million/year (Bilimoria *et al.* 2008). ACC exhibits a bimodal distribution predominantly affecting women (55–60%) and presenting in pediatric and adult populations. Approximately 75% of cases involve adrenal hormonal hypersecretion, primarily of cortisol and androgen, while estrogen or mineralocorticoid excess is rare (Abiven *et al.* 2006).

Due to the rarity of ACC, limited preclinical models and clinical trials have hindered progress, resulting in poor outcomes, with a 5-year survival rate of 35% (Abiven *et al.* 2006) dropping to less than 15% in metastatic stage (Kerkhofs *et al.* 2013). Nevertheless, more recent studies reported a slight increase in survival rates, being RO resection cornerstone of curative treatment for ACC. In this regard, estimated 5-year overall survival (OS) for ACC patients undergoing R0 resection was 64.8% compared to 33.8% for patients undergoing an R1 resection (Anderson *et al.* 2018). Prognosis worsens with advanced age and cortisol hypersecretion (Abiven *et al.* 2006). ENSAT (European Network for the Study of Adrenal Tumors) staging is currently the most reliable prognostic tool (Fassnacht *et al.* 2018).

Surgery is the only curative treatment for ACC, with adrenalectomy being the standard approach for localized ACC (Fassnacht *et al.* 2018). Systemic therapies, including chemotherapy, radiotherapy and mitotane, provide limited efficacy in unresectable or metastatic ACC. Advances in molecular research have identified key drivers such as insulin-like growth factor 2 (*IGF2*), β-catenin (*CTNNB1*) and *TP53*, with integrated genomic analyses revealing distinct molecular subgroups with varied prognoses (Giordano *et al.* 2009, Assié *et al.* 2014, Zheng *et al.* 2016).

This article provides a multidisciplinary update on the diagnosis, management and follow-up of ACC, incorporating contributions from experts in endocrinology, medical and radiotherapeutic oncology, pathology, interventional radiology, urology and surgery. The consensus, coordinated by the Spanish Society of Endocrinology and Nutrition (SEEN) and the Spanish Group of Neuroendocrine and Endocrine Tumors (GETNE), includes contributions of experts belonging to related societies: Spanish Association of Surgeons (AEC), Spanish Society of Urology (AEU), Anatomic-Pathology (SEAP), Medical Oncology (SEOM), Nuclear Medicine (SEMNIM) and Radiotherapeutic Oncology (SEOR). To reach this consensus, the experts of different specialties conducted a review of the literature, analyzed it according to the GRADE (grading of recommendation, assessment, development and evaluation) methodology and made proposals for guidelines, which were rated by other experts. Only the expert opinions with strong agreement were selected.

## Diagnostic approach to ACC: hormonal, genetic, radiological and functional (theragnostic) imaging evaluation

Given the aggressive nature of ACC, a prompt and comprehensive diagnosis approach combining clinical, hormonal and radiological assessment by multidisciplinary expert center team is crucial for accurate staging and determining surgical resectability, which can improve patient outcomes (Fassnacht *et al.* 2011).

### Radiological evaluation

**Figure 1:**
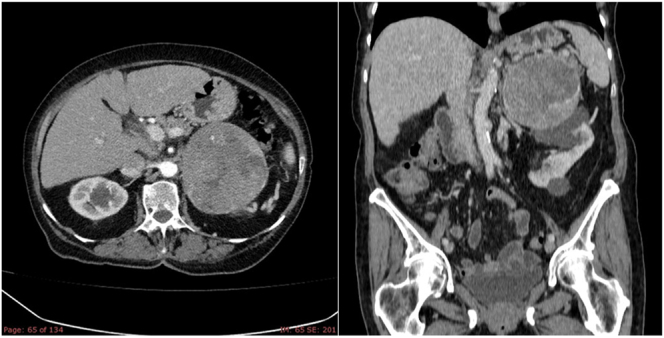
Radiological features of an ACC. CT of the abdomen with intravenous iodinated contrast with abdominal acquisition in arterial and portal phases: solid left adrenal mass with heterogeneous enhancement (8.2 × 9 × 9.3 cm, CCxTxAP). It displaces the tail of the pancreas cranially and caudally to the ipsilateral kidney. It does not show macroscopic fat foci or calcifications. The findings suggest adrenal carcinoma or pheochromocytoma as the first possibility. Pathological retroperitoneal lymphadenopathy (left paraaortic and interaortocaval) and in the hepatic hilum.

Radiological imaging plays a pivotal role in staging, evaluating disease spread and assessing the surgical feasibility. The primary imaging modalities include (Fassnacht *et al.* 2023*b*):

**Computed tomography (CT)** is the first-line imaging modality for adrenal masses due to its resolution, speed and accessibility (Ilias *et al.* 2007). ACC typically presents as a large (often >4 cm), irregular mass with heterogeneous features (necrosis, hemorrhage or calcification) and low lipid content, resulting in high Hounsfield units (HU) (>10 on unenhanced CT) (Petersenn *et al.* 2015, Araujo-Castro *et al.* 2020, Mínguez Ojeda *et al.* 2022) (Fig. 1). Contrast-enhanced CT with multiphase imaging aids in evaluating vascularity and differentiating benign from malignant lesions. Some data that may be suggestive of malignancy include an absolute washout of less than 60% and a relative washout of less than 40% (Ahmed *et al.* 2020). However, some studies found that the sensitivity of these cut-offs of relative and absolute washout is low. For example, according to the Marty M series, sensitivity of these cut-offs of relative and absolute washout was 72.3% and 76.6%, respectively (Marty *et al.* 2018); and according to the Schloetelburg’s study (Schloetelburg *et al.* 2021), the established thresholds of 60% for absolute and 40% for relative washout misclassified 35.9% and 35.2% of the masses, respectively.

Staging evaluation assesses local invasion such as involvement of the renal vein, inferior vena cava (IVC) or hepatic vein, and distant metastases, primarily in the liver, lungs and lymph nodes. Liver metastases often necessitate a multiphase study, including arterial, venous and delayed phases, to ensure accurate detection (Ilias *et al.* 2007).

**Abdominal magnetic resonance imaging (MRI)** serves as a complementary or alternative tool, particularly when CT contrast is contraindicated, or findings are inconclusive. ACC appears as a heterogeneous mass with hyperintense T2-weighted signals (necrosis/hemorrhage) and hypointense regions on chemical shift imaging, distinguishing lipid-poor adenomas from ACC. MRI is also valuable in assessing venous invasion (IVC or renal veins) and provides enhanced tissue characterization compared to CT (Byung *et al.* 2007, Shin & Kim 2015).

### Hormonal and genetic evaluation

In addition to standard hormonal evaluation for adrenal incidentalomas, the ESE/ENSAT guidelines recommend measuring sex steroids and steroidogenesis precursors, ideally using multisteroid profiling by tandem mass spectrometry in cases where ACC is suspected based on imaging or clinical features (Table 1) (Fassnacht *et al.* 2023*b*). A complete preoperative endocrine work-up is necessary to establish the tumor’s secretory profile, identify biomarkers for recurrence, and optimize perioperative management (e.g., perioperative glucocorticoid therapy in patients with hypercortisolism). Key evaluations include measuring urinary or plasma normetanephrine and metanephrine to rule out pheochromocytoma and prevent intraoperative complications. Cortisol excess should be assessed even in the absence of Cushingoid features to mitigate the risk of life-threatening postoperative adrenal insufficiency in cortisol-secreting ACCs. Aldosterone-secreting tumors should be managed preoperatively to address hypertension and hypokalemia (Gaujoux *et al.* 2017*a*, Puglisi *et al.* 2018*a*).

**Table 1:** Hormonal evaluation in patients with suspected ACC (Fassnacht *et al.* 2018).

Designation purpose	Recommended test
Assessment of glucocorticoid secretion disorders	- 1 mg dexamethasone suppression test - 24 h urinary free cortisol - ACTH levels
Assessment of mineralocorticoid secretion disorders	- Serum potassium levels - Aldosterone-to-renin ratio (for hypertensive patients)
Evaluation of sex hormones and steroid precursors	- DHEA-S, 17-hydroxyprogesterone, androstenedione - Testosterone (for women only), 17β-estradiol (for men and postmenopausal women) - 11-Deoxycortisol
Exclusion of pheochromocytoma	- 24 h urinary fractionated metanephrines and/or plasma-free metanephrines

For all adult patients with ACC, at least a basic clinical genetic evaluation exploring personal and family history for evidence of hereditary predisposition syndrome should be carried out to identify potential hereditary predisposition syndromes (Fassnacht *et al.* 2018). Germline genetic evaluation should be performed in those patients with clinical and/or family history suggestive of hereditary disorders. Detecting germline mutations impacts patient care and surveillance while allowing identification of at-risk relatives (Fassnacht *et al.* 2018). The most frequent syndromes associated with ACC in adults are Li Fraumeni (LFS) and Lynch syndromes in 5% and 3% of cases, respectively (Herrmann *et al.* 2012, Raymond *et al.* 2013). Nonetheless, LFS accounts for 50–80% of pediatric ACCs (Bougeard *et al.* 2008). Lynch syndrome screening involves immunohistochemistry for *MSH2*, *MLH1*, *PMS2*, *MSH6* and microsatellite instability testing, or direct germline analysis of these genes and *EPCAM*. LFS diagnosis relies on detecting pathogenic variants in *TP53* (Petr & Else 2016). Less frequent genetic syndromes associated with ACC include Beckwith–Wiedemann syndrome (children), familial adenomatous polyposis (APC), Carney complex and MEN1 (Fassnacht *et al.* 2018).

### Functional (theragnostic) imaging evaluation

Nuclear imaging, specifically positron emission tomography with 18F-fluorodeoxyglucose ([18F]FDG PET/CT), has become integral in the diagnosis, staging and management of ACC. This technique helps in distinguishing benign from malignant adrenal lesions and correlates metabolic activity with tumor aggressiveness (Fassnacht *et al.* 2018). Emerging theragnostic agents, such as fibroblast activation protein inhibitors (FAPI) and C-X-C motif chemokine receptor 4 (CXCR4)-directed radiotracers, hold promise for combining diagnostic imaging with targeted radionuclide therapies, advancing precision medicine in ACC.

#### [18F]FDG PET/CT in ACC

ACC typically exhibits high metabolic activity, detectable on [18F]FDG PET/CT as areas of increased glucose uptake, correlating with malignancy markers such as Ki67. An adrenal-to-liver SUVmax ratio >1.5 demonstrated 100% sensitivity for malignancy and 87% specificity, establishing it as a robust diagnostic indicator (Libé *et al.* 2023, Romanisio *et al.* 2024), excluding pheochromocytoma. Furthermore, [18F]FDG PET/CT aids in evaluating metastatic spread and guiding surgical decisions by providing a more comprehensive disease overview (Table 2). However, we should be aware that although FDG-PET/CT has the advantage of the low risk of false negative results (namely missing a malignant adrenal tumor), it is clearly not zero, and several benign adrenal tumors (e.g., functional adenomas) may be FDG-positive lesions (Fassnacht *et al.* 2023*b*, Libé *et al.* 2023).

**Table 2:** Clinical applicability of the [18F]FDG PET/CT in the diagnosis of ACC.

Clinical scenario	Description
Diagnostic accuracy of [18F]FDG PET/CT (Ma *et al.* 2021)	The specificity and sensitivity of [18F]FDG PET/CT for ACC are high, with several studies recommending an SUVmax cut-off value around 5.65 for differentiating ACC from benign adrenal lesions. The quantification of uptake values, such as the SUVmax and adrenal-to-liver ratio, is crucial for this differentiation
Correlation with tumor aggressiveness and Ki67 (Libé *et al.* 2023, Ma *et al.* 2021, Romanisio *et al.* 2024)	In tumors with a Ki67 index exceeding 10%, [18F]FDG uptake tends to be markedly elevated, suggesting a more aggressive disease course and a potentially poorer prognosis
Use in differential diagnosis of adrenal lesions (Ma *et al.* 2021)	In a comparative study, malignant lesions showed a SUVmax mean of 10.0, compared to 5.4 in benign lesions, indicating a significant threshold for malignancy risk assessment. The T/L ratio, or tumor-to-liver ratio, is another useful metric, with malignant lesions often exhibiting ratios >3, in contrast to benign cases, which generally fall below this threshold

#### Theragnostic radiotracers in ACC management

Theragnostic represents a transformative approach to ACC management by combining diagnosis imaging with targeted treatment. FAPI and CXCR4-directed agents, such as [68Ga]Ga-pentixafor, exemplify this paradigm, identifying patients for radionuclide therapies and improving outcomes.

FAPI target cancer-associated fibroblasts within the tumor microenvironment present in ACC. FAPI PET/CT has demonstrated high specificity in visualizing tumor-associated stroma, enabling clearer delineation of ACC lesions (Chopra *et al.* 2023). Moreover, FAPI agents conjugated with therapeutic radionuclides, such as [177Lu]Lu, allow for high-dose radiation directly to the tumor with minimal damage to surrounding tissues, offering a promising approach for recurrent or metastatic ACC (Michalski *et al.* 2023).

CXCR4 is implicated in tumor progression and metastasis in ACC. Its expression, detectable via [68Ga]Ga-pentixafor PET/CT, predicts poor outcomes, linking high CXCR4 expression with shorter OS (Dreher *et al.* 2024, Schloetelburg *et al.* 2024). Patients with CXCR4-positive tumors demonstrated an average survival of 6.4 months compared to 13.3 months in those with lower CXCR4 expression, highlighting its prognostic potential (Schloetelburg *et al.* 2024).

Radionuclide therapy is effectively used for diagnosis and treatment in various cancers, including neuroendocrine neoplasms, and specific radiotracers for ACC are available. Metomidate, an inhibitor of 11-beta-hydroxylase (CYP11B), has been labeled with 11C, 18F and 131I for SPECT and SPECT/CT scanning, showing good sensitivity and specificity. Other radiotracers that bind CYP11B enzymes include 18F-fluoroetomidate (FETO) and 123I-iodometomidate (IMTO) (Wong *et al.* 2016). A case series involving 11 patients treated with 123I-IMTO reported one partial response and five stable diseases, with a median progression-free survival (PFS) of 14 months for responding patients and an overall median survival of 13 months for the cohort (Hahner *et al.* 2012). Another CYP11B-ligand tracer, 131I-IMAZA, has been tested in 13 refractory advanced ACC patients without responses and a PFS of 14 months, warranting cautious interpretation of results (Hahner *et al.* 2022).

## Pathological and molecular markers of prognosis

The rarity of ACC complicates its histopathological differentiation from benign lesions and other neoplasms. Pre-surgical adrenal biopsy is unnecessary when surgery is not an option. Advances in histopathology, ancillary studies and genetics, as reflected in the 2022 WHO classification, have improved diagnostic accuracy (Mete *et al.* 2022). The modified Weiss scoring system (Weiss *et al.* 1989) is the most widely used tool, requiring at least three of nine histological parameters for malignancy: high nuclear grade, >5 mitoses/50 high-power fields, atypical mitosis, >75% eosinophilic cytoplasm, >33% diffuse architecture, necrosis or vascular/sinusoidal/capsular invasion.

In pediatric ACC, conventional adult criteria may lead to overdiagnosis of malignancy. The Wieneke score system (Wieneke *et al.* 2003) provides pediatric-specific parameters, including tumor weight >400 g, size >10.5 cm, local tissue or organ invasion and a high mitotic index, requiring at least four criteria for diagnosis.

Cytopathological variants of ACC include conventional (eosinophilic or lipid-rich), oncocytic, myxoid or sarcomatoid forms. The oncocytic variant, the most common, is characterized by high-grade nuclei and relies on the Lin–Weiss–Bisceglia system (Bisceglia *et al.* 2004) for malignancy diagnosis, requiring at least one major criterion: >5 mitoses/50 HPF, atypical mitoses or venous invasion.

Immunohistochemistry is essential for confirming ACC and excluding non-adrenal cortical origins. Key markers include SF1, synaptophysin, melan A and inhibin alpha, with SF1 being the most reliable (Bisceglia *et al.* 2004). High-grade ACC often exhibits molecular alterations, such as IGF2 overexpression, p53 mutations or loss (Mete *et al.* 2018) and β-catenin accumulation (Borges *et al.* 2020).

## Surgical treatment

### Indications of surgery

Complete surgical resection is the only curative treatment for patients with localized ACC. The goal of surgery is to achieve an R0 resection, defined as complete tumor with microscopically negative margins. *En bloc* resection, including involved organs, is essential to avoid capsular disruption (Chagpar *et al.* 2014, Fernandez Ranvier & Inabnet 2015, Memeh *et al.* 2025). Five-year survival rates range from 30 to 50%, while incomplete resection or metastatic disease reduces the median survival to less than 1 year (Fernandez Ranvier & Inabnet 2015, Sinclair *et al.* 2020, Kwon *et al.* 2024). Surgical resection is the definitive treatment for stages I–III, including tumors with local invasion into surrounding organs or IVC. Stage III tumors initially deemed unresectable may become resectable following partial response to neoadjuvant therapy. The role of surgery in stage IV metastatic disease is highly individualized (Sinclair *et al.* 2020).

### Principles of surgical treatment for localized stages (ENSAT stages I-III)

Curative treatment requires complete surgical resection with negative margins. Open adrenalectomy remains the gold standard due to superior oncologic outcomes, particularly in cases requiring *en bloc* resection of adjacent structures or extensive lymphadenectomy (Datta & Roses 2016, Kastelan *et al.* 2021, McCoy *et al.* 2022, de Ponthaud *et al.* 2024).

**Figure 2:**
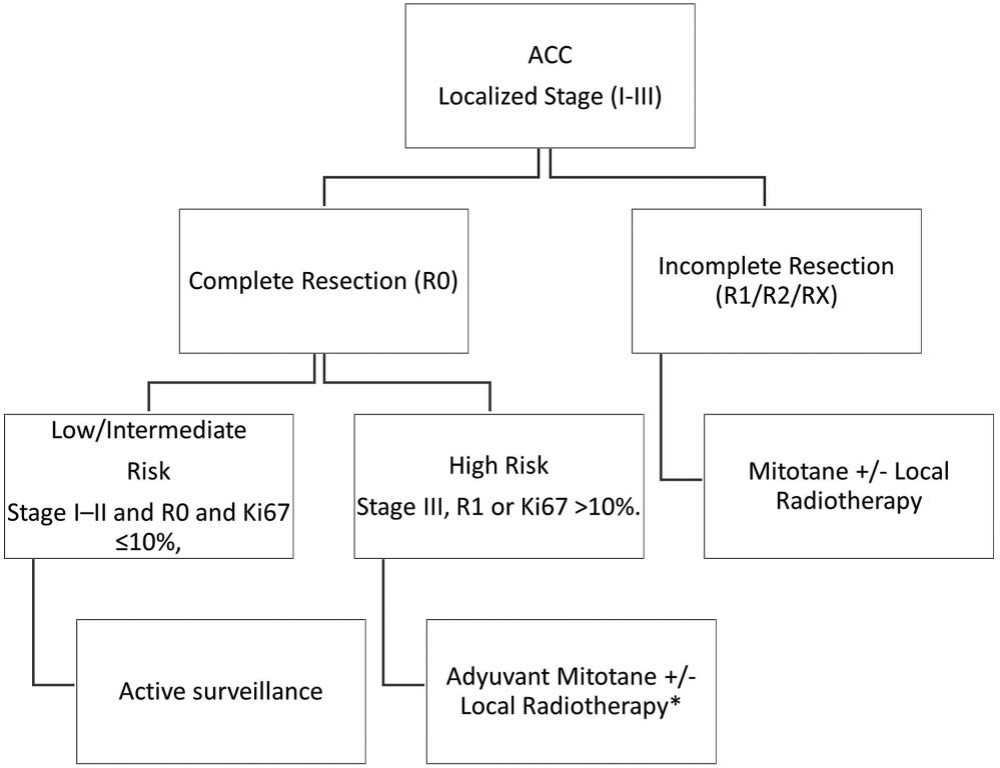
Surgery in localized ACC (stage I–III ENSAT). ACC, adrenocortical carcinoma. *Adjuvant chemotherapy may be considered in selected patients with very high risk for recurrence.

The deep retroperitoneal location, hypervascular attachments to adjacent organs and fragile capsule of the adrenal glands require meticulous surgical technique to minimize the risk of tumor rupture. These anatomical challenges reinforce the recommendation for open adrenalectomy as the preferred approach, particularly for achieving adequate exposure and optimal oncologic outcomes (Fig. 2) (Gaujoux *et al.* 2017*a*).

Several studies have defended the superiority of open adrenalectomy over laparoscopic approaches, with higher rates of complete oncologic resection and improved surgical outcomes (Maurice *et al.* 2017, Dickson *et al.* 2018). In addition, Hu X meta-analysis (Hu *et al.* 2020) that included 15 studies incorporating 2,207 patients with ACC found that minimally invasive adrenalectomy surgery (MIS) approaches were likely to have a better recovery, but were associated with earlier recurrence and more positive surgical margin and peritoneal recurrence. Consequently, the Society of Surgical Oncology (SSO), the European Society of Endocrine Surgeons (ESES) and ENSAT strongly recommend open adrenalectomy as the gold standard for confirmed or highly suspected ACC and evidence for local invasion. However, for tumors <6 cm without any evidence of local invasion, laparoscopic adrenalectomy (respecting the principles of oncological surgery) is reasonable if the surgeon has good expertise (Supplementary Material S1 (see section on Supplementary materials given at the end of the article)) (Lombardi *et al.* 2012, Fassnacht *et al.* 2018).

In experienced centers, laparoscopic adrenalectomy may be acceptable for suspicious lesions smaller than 8–10 cm without pre- or perioperative evidence of local invasion (stages I–II). However, oncologic principles must be strictly followed, including complete resection with negative surgical margins through en bloc removal of peri-adrenal and retroperitoneal fat, maintenance of capsular integrity by minimizing gland manipulation to prevent rupture or fragmentation and routine performance of regional lymphadenectomy (Leboulleux *et al.* 2010, Gaujoux & Brennan 2012, Lombardi *et al.* 2012, Cooper *et al.* 2013, Donatini *et al.* 2014, Fassnacht *et al.* 2018, Wu *et al.* 2018, Ginsburg *et al.* 2022).

### Surgical treatment for recurrent and/or advanced ACC

**Figure 3:**
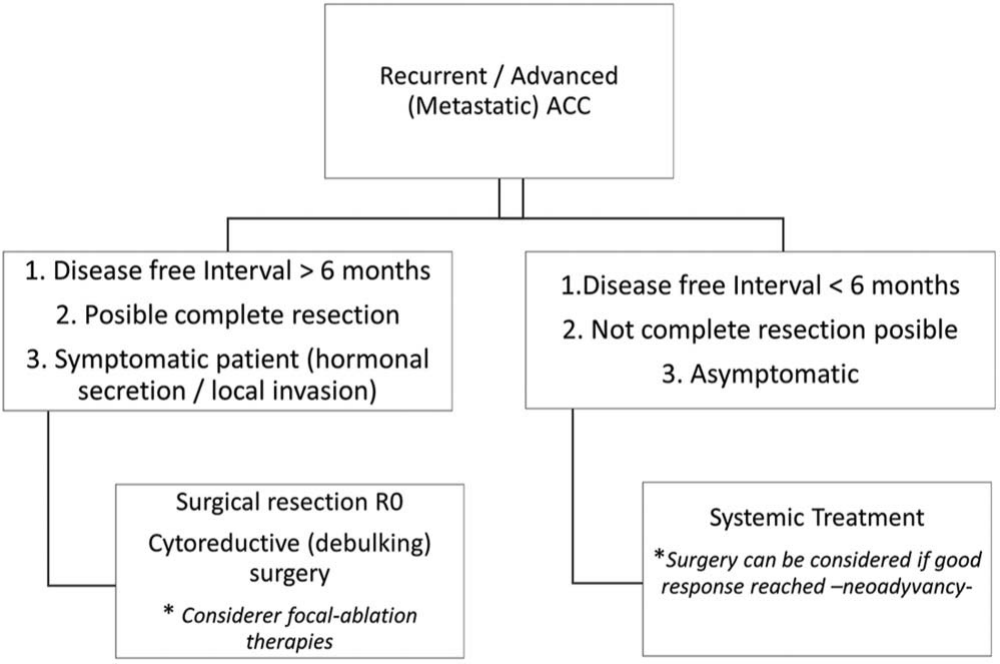
Surgery in advanced ACC (stage IV ENSAT). ACC, adrenocortical carcinoma.

Figure 3 shows the algorithm for the management of the patient with recurrent or metastatic ACC. Up to 21–39% of patients with ACC presented with oligo- or multi-metastatic disease at diagnosis. Approximately 80% of those who undergo complete resection will have local or distant recurrence (Datta & Roses 2016, Gaujoux *et al.* 2017*b*). However, the rate of recurrence is lower in more recent series. For example, in the study by Puglisi *et al.* (Puglisi *et al.* 2023*b*) that included 512 patients with ACC, recurrence free survival was 59% (95% CI, 43–80) for stage I ACC, 36% (95% CI, 29–44) for stage II and 16% (95% CI, 9–27) for stage III.

While data on surgical treatment in metastatic ACC are scarce, evidence suggests that complete resection of primary lesion and metastases can improve outcomes when technically feasible (Erdogan *et al.* 2013, Fassnacht *et al.* 2018, Terzolo *et al.* 2023). A retrospective study demonstrated an increase in the median OS (28.6 vs 13.0 months) at 1 (69.9 vs 53.0%) and 2 (46.9 vs 22.1%) years for patients undergoing complete resection (Livhits *et al.* 2014). Neoadjuvant chemotherapy has been shown to enhance 5-year survival (41.7 vs 8.9%) and can help to select patients who are suitable for multimodal management, excluding those with rapidly progressive, chemotherapy-resistant disease (Erdogan *et al.* 2013, Livhits *et al.* 2014, Kenney & Hughes 2023). However, it should be noted that data on neoadjuvant chemotherapy in the treatment of ACC is limited by few existing trials, most of which are retrospective. In this regard, the current consensus for its use is to reduce the burden of disease to facilitate complete resection (Kenney & Hughes 2023). In selected cases, particularly in patients with severe hormone excess, debulking surgery might be an option, if >80% of tumor burden can be safely removed (Erdogan *et al.* 2013, Fassnacht *et al.* 2018, Terzolo *et al.* 2023). For patients with poor clinical condition or localized metastatic burden, focal ablative therapies may serve as an alternative (Wood *et al.* 2003, Cazejust *et al.* 2010, Ho *et al.* 2013).

Complete resection of recurrent ACC has been associated with improved PFS and OS. Data from the German Adrenal Carcinoma Registry identified time to first recurrence >12 months as a positive prognostic factor. Surgery should be considered for patients with local or distant recurrence when time to recurrence is ≥6 months, and ideally ≥12 months (Erdogan *et al.* 2013, Livhits *et al.* 2014, Fassnacht *et al.* 2018). Another more recent study of Calabrese *et al.* (2023) in a series of 106 ACC patients that experienced recurrence described that 60.4% of patients became free of disease, attaining a second remission free survival of 15 months (IQR 6–64) after the treatment of recurrence. Margin status Rx (hazard ratio (HR) 2.62) and R1 (HR 4.37), percent increase in Ki67 (HR 1.03) and recurrence in multiple organs (HR 3.92) were associated with an increased risk of mortality, while adjuvant mitotane treatment (HR 0.30) and longer time to first recurrence (HR 0.93) were associated with reduced risk.

## Mitotane and other steroidogenesis inhibitors

### Mitotane

Mitotane (o,p′-DDD) is the sole agent that mainly targets adrenal tissue, playing a central role in ACC treatment due to its adrenolytic and anti-steroidogenic effects. It is used in the adjuvant setting for high-risk patients, as systemic therapy for metastatic disease and/or to control hormonal secretion (Allolio & Fassnacht 2006).

#### Mitotane in adjuvant setting

The ESE/ENSAT guidelines suggest adjuvant mitotane in patients with ACC who have undergone complete surgical resection and have a high risk of recurrence defined as Ki67 > 10%, ENSAT stage III or IV or Rx-R1 resection (Fassnacht *et al.* 2018). The ADIUVO trial (Terzolo *et al.* 2023), the first randomized study evaluating adjuvant mitotane in low- to intermediate-risk, showed no significant benefit in recurrence-free survival (RFS) or OS for patients with stage I–III ACC and Ki67 ≤ 10%. In relation to high-risk patients, Calabrese study (Calabrese *et al.* 2019), including 152 nonmetastatic ACC patients (100 treated with adjuvant mitotane and 52 patients were left untreated following surgery), showed a higher risk of recurrence (HR: 2.79, 95% CI: 1.58–4.91; *P* < 0.001) in not-treated patients compared to mitotane-treated group. In addition, they observed that adjuvant mitotane treatment reduced significantly the risk of death in patients with elevated Ki67 index (*P* = 0.005) and in patients with stage III ACC (*P* = 0.02).

In relation to the situation of adjuvant therapy with mitotane in Spain, it is worth noting the data from the ICARO-GETTHI/SEEN registry. A recent study that included 244 patients nonmetastatic, resectable ACC (TNM stages I-III) who underwent primary tumor resection, of whom 133 (52%) received adjuvant mitotane, indicates a 39% reduced recurrence risk (HR 0.61; 95% CI, 0.39–0.95) for mitotane-treated patients (Carmona-Bayonas *et al.* 2025). However, the effects have diminished over 24 months. Thus, the Spain data suggests that adjuvant mitotane delays recurrence, but yet questions remain as to its curative capacity.

Adjuvant mitotane is typically initiated within 6–8 weeks post-surgery, and continued long-term, often exceeding 2 years, depending on tolerance. Continuous treatment is advised until there is evidence of disease progression or unacceptable toxicity (Fassnacht *et al.* 2020*a*, 2023*a*). However, some studies found that the survival curves of patients treated up to 24 months vs patients treated for a longer period, both for RFS and RFS after mitotane, did not show any significant difference (Basile *et al.* 2021).

#### Mitotane in patients with metastatic disease

For metastatic ACC, mitotane is utilized as monotherapy or in combination with chemotherapy, depending on the individual patient’s needs and treatment goals (Fassnacht *et al.* 2020*a*, 2023*a*). As a single agent, it provides symptomatic relief and stabilizing disease in patients unsuitable for combination regimens particularly for those with low tumor burden and a low Ki67 index. Moreover, objective responses in patients with metastatic ACC have been described. For example, Megerle’s study including 127 patients with advanced ACC treated with mitotane monotherapy found that 26 patients (20.5%) experienced objective response, including three with complete remission (Megerle *et al.* 2018). When combined with chemotherapy, mitotane enhances outcomes by leveraging its adrenolytic properties alongside cytotoxic effects of chemotherapy (phase III FIRM-ACT trial) (Fassnacht *et al.* 2020*a*, 2023*a*). The use of mitotane in combination with locoregional therapies is another option in low volume metastatic ACC patients. In this regard, a retrospective study of 79 patients with stage IV ACC, with two or fewer tumoral organs who received mitotane (19 in monotherapy and 60 in combination with locoregional therapies (LRT)), showed that OS was statistically longer in the mitotane plus LRT group compared to the mitotane-only group (HR 0.27; 95% CI, 0.14–0.50). In addition, ten (13%) patients achieved complete response, all from the mitotane plus LRT group (Boilève *et al.* 2021).

#### Mitotane for hormonal secretion control

Mitotane inhibits steroidogenesis by targeting enzymes such as 11β-hydroxylase, reducing cortisol production (Fassnacht *et al.* 2018) and alleviating symptoms of hormone excess, including hypertension, weight gain and glucose intolerance (Del Rivero *et al.* 2024). Moreover, a relevant effect of mitotane on hormonal control is due to the induction of cytochrome P450 3A4 (CYP3A4) and through increased levels of corticosteroid-binding globulin (CBG) (Chortis *et al.* 2013*a*).

For mild hormone secretion, mitotane alone is generally sufficient; although its therapeutic effects typically take several weeks to become evident, severe cases may require additional measures (Fassnacht *et al.* 2018).

#### Dosing, monitoring and adverse effects

Mitotane dosing varies based on performance status and mostly on the patient’s and physician’s preferences. However, in general, high-dose regimens should be considered for robust patients and low-dose regimens for those with poorer tolerance (De Filpo *et al.* 2021, Fassnacht *et al.* 2025) (Table 3).

**Table 3:** Dosing strategies for initiating mitotane therapy (Fassnacht *et al.* 2025).

	Day 1	Day 2	Day 3	Day 4	Post day 4
High-dose regimen	1.5 g/day	3.0 g/day	4.5 g/day	6.0 g/day	Measure blood levels in 2–3 weeks
Low-dose regimen	1.0 g/day			1.5 g/day	Continue increasing by 0.5 g/day every 3–4 days up to 3.0–4.0 g/day; adjust dose based on mitotane levels and tolerability

Plasma levels should be maintained above 14 mg/L for efficacy, while levels above 20 mg/L should be avoided due to toxicity risks. Monitoring begins every 3–4 weeks during dose titration and is spaced to 6–12 weeks once levels stabilize (Allolio & Fassnacht 2006). Table 4 describes frequency of adverse effects with mitotane therapy and Table 5 provides some recommendations for monitoring these adverse events.

**Table 4:** Secondary adverse effects with mitotane.

Very common ≥1/10 patients	Common ≥1/100 to 1/10	Rare ≥1/10,000 to <1/1,000
Gastrointestinal Adrenal insufficiency Increase in hepatic enzymes Hepatic microsomal enzyme induction Increase in hormone-binding globulins disturbance of thyroid parameters hypercholesterolemia, hypertriglyceridemia Other: gynecomastia	CNS Hematological: prolonged bleeding time Leucopenia Primary hypogonadism in men Dermatological	Liver failure, autoimmune hepatitis Hematological: thrombocytopenia, anemia

CNS, central nervous system.

**Table 5:** Recommendations for monitoring adverse effects of mitotane.

Type of adverse-effect	Potential adverse effects	Monitoring parameters	Frequency of monitoring
Gastrointestinal	Nausea, vomiting, diarrhea, anorexia	Symptoms diary, nutritional status	Weekly initially, then as needed
Adrenal	Adrenal insufficiency	Serum cortisol levels, ACTH levels	Every 2–4 weeks during titration
Central nervous system	Lethargy, somnolence, vertigo, ataxia, confusion, depression, dizziness, decreased memory	Neurological assessments, patient reports	Monthly or as symptoms arise
Hepatic	Increased hepatic enzymes, liver failure	Increased hepatic enzymes, liver failure	Increased hepatic enzymes, liver failure
Hormonal	Disturbance in thyroid parameters, increased hormone-binding globulins	Thyroid function tests, hormone levels	Every 3 months
Metabolic	Hypercholesterolemia, hypertriglyceridemia	Lipid panel	Every 3 months
Dermatological	Skin rash	Visual skin assessments	As needed, based on patient reports
Hematological	Leucopenia, thrombocytopenia, anemia, prolonged bleeding time	Complete blood count	Every 2–4 weeks initially, then every 3 months
Gynecomastia	Development of gynecomastia	Patient reports, physical examination	At each follow-up visit

Adverse events must be carefully managed, including hydrocortisone replacement, which requires high-dose adjustments (2-3×) due to mitotane-induced cytochrome P450 3A4 activation (Kroiss *et al.* 2011), which accelerates hydrocortisone metabolism and increases cortisol-binding protein (CBG) levels (Chortis *et al.* 2013b).

### Other steroidogenesis inhibitors used in ACC

Additional inhibitors, including metyrapone, osilodrostat, ketoconazole and etomidate, are used for hypercortisolism management (Supplementary Material S2) (Varlamov *et al.* 2021). Combination regimens or ‘block-and-replace’ approaches with hydrocortisone may be required for rapid cortisol reduction, particularly before surgery or chemotherapy or in advanced disease (Turla *et al.* 2022). Severe hypercortisolism requires anticoagulation and pneumocystis prophylactic antibiotics until cortisol levels are controlled (Nieman *et al.* 2015).

In cases of aldosterone-secreting tumors causing hypertension and hypokalemia, mineralocorticoid receptor antagonists such as spironolactone or eplerenone combined with potassium supplementation and electrolyte monitoring are mandatory.

## Locoregional therapies

For unresectable or metastatic disease, LRT are recommended by international guidelines (Bechmann *et al.* 2020, Fassnacht *et al.* 2018, 2020*b*). Although ACC’s rarity and aggressive nature limit large prospective studies, evidence from retrospective analyses suggests that LRT can provide tumor control, symptom relief and potentially prolonged survival (Veltri *et al.* 2020). Available options include percutaneous thermal ablation (PTA), bland transarterial embolization (TAE), transarterial chemoembolization (TACE), selective internal radiation therapy with yttrium-90 microspheres (SIRT) and radiotherapy (Table 6).

**Table 6:** Comparison of TAE, TACE and SIRT in metastatic ACC.

Aspect	TAE (transarterial embolization)	TACE (transarterial chemoembolization)	SIRT (selective internal radiation therapy)
Mechanism of action	Embolization of blood supply to tumor	Combines chemotherapy with embolization	Delivers localized radiation (yttrium-90 microspheres)
Procedure	Injects embolic agents to block arteries	Injects chemotherapy drugs + embolic agents	Radioactive microspheres injected into hepatic arteries
Primary effects	Ischemic necrosis of tumor	Cytotoxic chemotherapy + ischemic necrosis	Radiation-induced DNA damage and tumor necrosis
Patient selection	Suitable for patients who cannot tolerate chemo	Limited by number and size of liver lesions	Not limited by number and location of metastases
Procedure frequency	Repeatable if tolerated	Often requires multiple sessions	Generally one-time per liver lobe
Side effects	Post-embolization syndrome (pain, fever)	Post-embolization syndrome + chemo-related si adverse de effects	Transient transaminase increases, mild fatigue, nausea and pain
Limitations	Less effective for large hypervascular tumors	Not for large diffuse disease or vascular invasion	Requires preserved liver function

### Radiotherapy

The role of adjuvant radiotherapy in ACC remains controversial. Retrospective studies and older radiotherapy techniques contribute to the uncertainty surrounding its efficacy. However, it might benefit patients at higher risk of relapse, particularly when combined with mitotane in cases of R1 resection, uncertain margins or stage III disease (Ho *et al.* 2013).

A pooled analysis of four studies in the 2018 ESE/ENSAT guidelines showed mixed results (Fassnacht *et al.* 2018), with a HR of 0.8 (95% confidence interval (CI): 0.6–1.1) for recurrence and 1.0 (95% CI: 0.7–1.5) for mortality, highlighting the limitations of observational data (Fassnacht *et al.* 2006, Habra *et al.* 2013, Else *et al.* 2014, Sabolch *et al.* 2015).

Recent studies, such as a meta-analysis by Zhu *et al.* (2020), demonstrated higher OS and locoregional recurrence- and disease-free survival with radiotherapy. Khosla *et al.* (2023) identified capsular invasion and positive margins as independent prognostic factors, with only three of 25 experienced local relapses after adjuvant radiotherapy.

Current evidence supports radiotherapy as a safe option to reduce local relapse risk, especially in high-risk patients, although prospective trials are needed to determine its effect on OS. The recommended treatment dose is 50–60 Gy to the tumor bed, with fractionation of 2 Gy per session.

### Other locoregional therapies

**Image-guided PTA techniques** including radiofrequency ablation (RFA), cryoablation, microwave ablation (MWA), irreversible electroporation and laser or external energy-based ablation are effective for controlling primary and metastatic lesions (liver, adrenal glands, kidneys, lungs and bones), particularly those <5 cm. A retrospective study of 66 patients with metastatic ACC reported a 20.5% complete remission rate in treated lesions, with a favorable safety profile (Kimpel *et al.* 2024). In the Veltri series with 32 patients with oligometastatic ACC (liver and lung metastases) who underwent image-guided ablation, complete ablation was obtained in 97% (29/30) and during follow-up, local tumor progression was registered in 7/29 cases (24.1%), with a median local tumor PFS of 21 ± 12.6 months (Veltri *et al.* 2020).

**Liver-directed therapies**, such as TAE, TACE and SIRT, offer tumor control and symptom relief in patients with liver-dominant metastatic ACC. A retrospective study of 65 patients showed significantly longer OS in those treated with TACE or SIRT compared to those without liver-directed therapy (32.4 vs 9.9 months; *P* = 0.011) (Owen *et al.* 2019).

In oligometastatic or slowly progressing ACC, combining LRT modalities can prolong time to progression (tTTP) and delay the need for systemic therapy. A study of 132 metastatic lesions found that favorable factors for prolonged tTTP included fewer prior treatments, higher mitotane plasma levels and smaller metastases (<3 cm) (Roux *et al.* 2022).

Further research is crucial to establish standardized LRT protocols, refine patient selection criteria and improve outcomes. Combining LRT with novel immunotherapies or targeted treatments may enhance outcomes. Personalized, multidisciplinary approaches are essential to optimize treatment decisions based on tumor location, local expertise and patient preferences.

## Chemotherapy

Currently, no clinical trial evidence supports adjuvant chemotherapy for ACC in adults. A multicenter ENSAT case–control study of 299 patients with resected ACC suggested potential benefits of platinum-based adjuvant chemotherapy for OS (HR 0.25, 95% CI: 0.09–0.69; *P* = 0.007) and RFS (HR 0.45, 95% CI: 0.29–0.89; *P* = 0.021) (Kimpel *et al.* 2021). In contrast, a retrospective analysis of the National Cancer Database including 577 patients with localized ACC reported no survival advantage from adjuvant chemotherapy in subgroups with lymphovascular invasion, positive margins or T3 tumors (Al Asadi *et al.* 2021). The ongoing phase III ADIUVO2 trial (NCT03583710) is evaluating 2 years of adjuvant mitotane with or without 3 months of cisplatin and etoposide in high-risk patients (stage I–III, Ki67 > 10%) (Sarvestani *et al.* 2023). In pediatric ACC, the ARAR0332 study assessed 78 patients with ACC across different stages, using adrenalectomy alone for stage I, adrenalectomy with retroperitoneal lymph node dissection for stage II and mitotane with neoadjuvant chemotherapy for stages III and IV. Five-year event-free survival rates for stages I, II, III and IV were 86.2, 53.3, 81 and 7.1%, respectively, with corresponding OS rates of 95.2, 78.8, 94.7 and 15.6% (Rodriguez-Galindo *et al.* 2021). Notably, stage III patients who received neoadjuvant treatment demonstrated improved outcomes compared to stage II patients managed with surgery alone, although extrapolation to adults remains uncertain.

**Figure 4:**
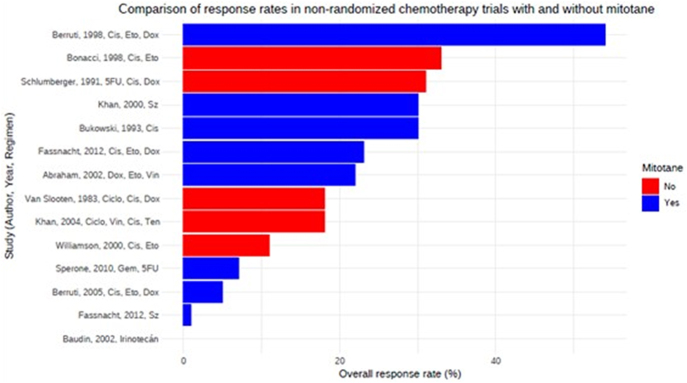
Comparison of response rates in non-randomized chemotherapy trials with and without mitotane in patients with ACC. A full color version of this figure available at https://doi.org/10.1530/ERC-25-0034.

Regarding advanced disease, combined chemotherapy regimens with mitotane are more active, although no direct comparisons exist (Fig. 4). Etoposide, doxorubicin and cisplatin combined with mitotane (EDP-M) remains the standard first-line therapy, as established by the phase III FIRM-ACT trial (Fassnacht *et al.* 2012). This trial randomized 304 patients to EDP-M or streptozotocin-mitotane (STZ-M), with crossover to the alternative regimen upon progression. EDP-M showed superiority in overall response rate (ORR; 23.2 vs 9.2%, *P* < 0.001) and PFS (5.0 vs 2.1 months; HR, 0.55; 95% CI: 0.43–0.69). OS was similar between groups (14.8 vs 12.0 months; HR, 0.79; 95% CI: 0.61–1.02). In the second-line setting, both regimens produced similar results to first-line therapy, with PFS-2 of 5.6 vs 2.2 months and OS-2 of 10.3 vs 7.4 months (95% CI: 6.3–9.2), favoring EDP-M. Serious adverse events occurred in 58% of EDP-M and 41% of STZ-M group (*P* = 0.16). For patients unable to tolerate EDP-M, platinum-etoposide is a common alternative, which demonstrated an ORR of 11% and OS of 10 months (Williamson *et al.* 2000). A phase II trial evaluating cisplatin and docetaxel did not demonstrate superiority over established regimens but may be a viable option for patients unable to receive etoposide or anthracyclines (Urup *et al.* 2013). The trial reported an ORR of 21%, a median PFS of 3 months (95% CI: 0.7–5.3) and an OS of 12.5 months (95% CI: 6–19), with neutropenia as the most common grade 3/4 toxicity (35%). In addition, continuous infusion doxorubicin, vincristine and etoposide combined with mitotane was investigated in a single-center phase II trial in 35 patients, both as first-line and subsequent therapy (Abraham *et al.* 2002). This regimen yielded an ORR of 22% and OS of 13.5 months. Prognostic factors included patient performance status and tumor functionality, with functional tumors linked to poorer outcomes.

Gemcitabine (GEM) with metronomic 5-FU or capecitabine (CAP), combined with mitotane, was evaluated in a phase II Italian trial involving 29 patients in the second- or third-line of treatment (Sperone *et al.* 2010). This regimen yielded an ORR of 7%, a disease control rate (DCR) of 46.3%, PFS of 5.3 months and OS of 9.8 months, with leukopenia as the most common grade III–IV toxicity (21.4%). In addition, a larger Italian-German cohort study of 145 patients explored clinical and molecular predictors of benefit from GEM-based chemotherapy (Henning *et al.* 2017). Concomitant mitotane levels above 14 mg/L and GEMCAP therapy were associated with prolonged PFS, while neither line of therapy nor *hENT1* or *RRM1* expression showed predictive value. Cabazitaxel was examined in an Italian phase II trial of 25 patients in the second or third line after progression on platinum-based therapy, showing no tumor responses, with a PFS of 1.5 months and OS of 6 months (Laganà *et al.* 2022).

A single-center prospective study of irinotecan in 12 patients treated in the second line showed no objective, clinical or biochemical responses and stabilization in 25% (Baudin *et al.* 2002). A phase II study in 11 patients with progressive metastatic ACC evaluated cyclophosphamide, doxorubicin and cisplatin (CAP), achieving an ORR of 18.2%, DCR of 72.7% and OS of 10 months (Van Slooten & Van Oosterom 1983). The OPEC regimen (vincristine, cisplatin, teniposide and cyclophosphamide) was evaluated in 11 Swedish patients after failure of streptozocin and mitotane (Khan *et al.* 2004). The 2-year OS rate was 82%, with an OS of 21 months, ORR of 18% and DCR of 82%. Although the regimen showed activity, significant adverse effects required dose adjustments.

In the Cosentini study (Cosentini *et al.* 2019) which included 28 patients with ACC, temozolomide achieved a DCR of 35.8%, an ORR of 21.4%, a PFS of 3.5 months and an OS of 7.2 months. The study reported a higher probability of ORR in patients with methylation of O6-methylguanine-DNA methyltransferase (MGMT) gene (50%) than in the non-methylated group (14.3%).

In summary, no clinical trial evidence currently supports adjuvant chemotherapy in adults, although the ADIUVO2 trial is ongoing, and data suggests the benefit of this approach in ACC with high-risk of recurrence. For advanced unresectable disease, EDP-M remains the standard first-line therapy, despite the lack of randomized trials comparing mitotane monotherapy with mitotane plus chemotherapy. Chemotherapy achieves disease control in approximately 30–50% of cases, but benefits are short-lived (median duration ∼5 months) with a median survival of around 1 year. In later lines, the GEMCAP regimen offers a favorable safety profile, although its efficacy remains limited (Table 7).

**Table 7:** Chemotherapy in advanced ACC (prospective studies).

Study	Year	Phase	Regimen	Mitotane	Line	N	ORR (%)	PFS (months)	OS (months)
Van Slooten & Van Oosterom (1983)	1983	II	CAP	No	Any	11	18	N/A	10
Williamson *et al.* (2000)	2000	II	EP	No	1st	37	11	N/A	10
Abraham *et al.* (2002)	2002	II	Dox, Eto, Vin	Yes	Any	35	11	N/A	13.5
Baudin *et al.* (2002)	2002	Prospective, but not clinical trial	Irinotecan	No	2nd	12	0	N/A	N/A
Sperone *et al.* (2010)	2010	II	GemFU/GEMCAP	Yes	2/3	28	7	5.3	9.8
Fassnacht *et al.* (2012)	2012	III	EDP	Yes	1st	151	23.2	5	14.8
					2nd	101		5.6	10.3
			Sz	Yes	1st	153	9.2	2.1	12
					2nd	84		2.2	7.4

N, number of patients; ORR, objective response rate; PFS, progression-free survival; OS, overall survival; N/A, not available/not reported; CAP, cyclophosphamide, doxorubicin and cisplatin; EP, etoposide and cisplatin; Dox, doxorubicin; Eto, etoposide; Vin, vincristine; GEMFU, gemcitabine and fluorouracil; GEMCAP, gemcitabine and capecitabine; EDP-M, etoposide, doxorubicin and cisplatin with mitotane; STZ, streptozotocin.

## Immunotherapy and immunocombinations

The clinical benefit of immune checkpoint inhibitors (ICIs) as second- or subsequent-line treatment in advanced ACC remains unsatisfactory (Araujo-Castro *et al.* 2021). A phase Ib trial with **avelumab**, including 50 pretreated patients (median two lines, range 1–6), showed an ORR of 6%, PFS of 2.6 months (95% CI: 1.4–4.0) and OS of 10.6 months (95% CI: 7.4–15.0), with a trend toward longer PFS in patients with *PD-L1* expression (Le Tourneau *et al.* 2018). In a phase II U.S. study with ten patients treated with **nivolumab** in second or subsequent lines, PFS was 1.8 months with predictable toxicity (Carneiro *et al.* 2019). A phase II single-center study (Memorial Sloan Kettering Cancer Center) with **pembrolizumab** in 39 patients showed an ORR of 23%, DCR of 52%, PFS of 2.1 months (95% CI: 2.0–10.7) and OS of 24.9 months (4.2-not reached) (Raj *et al.* 2020). *PD-L1* expression and *MSI-H/MMR-D* status did not correlate with response. Another phase II single-center study (MD Anderson Cancer Center) with pembrolizumab in 16 patients demonstrated a DCR of 36% at 27 weeks, ORR of 14% and DCR of 50% (Habra *et al.* 2019). Neither hormonal function, MSI status, nor *PD-L1* expression predicted response. A retrospective study with 54 patients treated with ICI across six German centers between 2016 and 2022 reported an ORR of 13.5%, DCR of 24%, PFS of 3.0 months and OS of 10.4 months (Remde *et al.* 2023). PD-L1 expression and nivolumab treatment compared to pembrolizumab were associated with longer survival.

The combination of **nivolumab and ipilimumab** in advanced ACC was assessed in a phase II multicohort trial of rare genitourinary cancers in 18 patients (McGregor *et al.* 2021). ORR was 6% and DCR was 53.3%, with a PFS of 4.5 months (95% CI: 1.8–6.6) and a 12-month PFS rate of 43% (95% CI: 8–75%). In the phase II Australian CA209-538 trial, the combination showed an ORR of 33% and a DCR of 66% (Klein *et al.* 2021). The phase 1/2 Spencer trial investigated **EO2401** (a cancer peptide therapeutic vaccine) combined with nivolumab in 33 patients treated in the first-line (21.2%) or subsequent lines (Baudin *et al.* 2022). The combination was well-tolerated. The ORR was 12%, DCR 24%, PFS 1.9 months (range 0.4–7.6) and the 6-month OS was 63%. In a post-hoc analysis, patients with clinical benefit had factors such as ECOG ≤1, diagnosis >9 months and ≤3 organs involved. In this group, the DCR was 64%, 6-month PFS 42% and 6-month OS 93%.

The combination of ICI with targeted therapy has been studied in several trials in pretreated patients. A phase II Chinese study with **camrelizumab and apatinib** in 21 patients showed an ORR of 52%, DCR of 95%, PFS of 12.6 months (95% CI: 8.4–20.9) and OS of 20.9 months (95% CI: 11.0–20.9) (Zhu *et al.* 2024). The phase II CABATEN trial, conducted by GETNE, included 24 advanced ACC patients treated with **atezolizumab and cabozantinib** after progression on chemotherapy and/or mitotane (Grande *et al.* 2024). ORR, the primary endpoint, was 8.3% (95% CI: 1–27), PFS was 2.9 months (95% CI: 2.8–5.7) and OS was 13.5 months (95% CI: 8.8–NR). Grade ≥3 adverse events occurred in 20.8% of patients, with hypertension (12.5%) and elevated transaminases (8.3%) being the most common. Despite limited activity, durable responses emphasize the need to explore predictive factors to optimize patient selection for this combination. In eight pretreated ACC patients of clinical practice, **pembrolizumab and lenvatinib** showed an ORR of 25%, DCR of 37.5% and PFS of 5.5 months (Bedrose *et al.* 2020).

**Figure 5:**
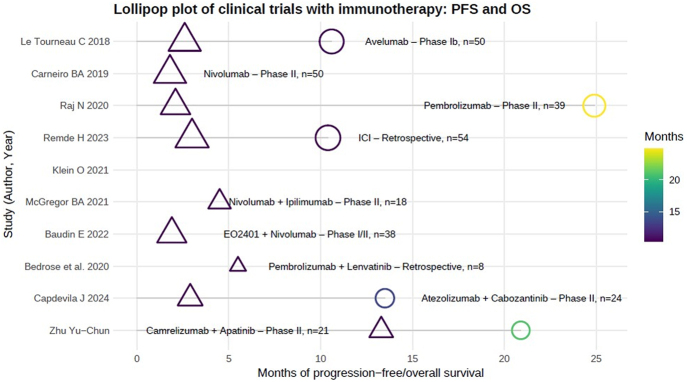
Lollipop plot of clinical trials with immunotherapy. PFS, progression-free survival; OS, overall survival. A full color version of this figure available at https://doi.org/10.1530/ERC-25-0034.

A key concern in treating functional ACCs with immunotherapy is potential resistance due to excessive glucocorticoid production (Araujo-Castro *et al.* 2021). To address this, a phase I trial combining relacorilant with pembrolizumab is ongoing (NCT04373265). A summary of studies with immunotherapy, either as monotherapy, dual immunotherapy or combined with targeted therapy in advanced ACC, is provided in Table 8 and Fig. 5.

**Table 8:** Trials with immunotherapy and immunocombinations (2018–2024).

Author, year	Phase	N	Treatment	Line	ORR (%)	PFS (months)	OS (months)
**Trials with immunotherapy in monotherapy**							
Le Tourneau *et al.* (2018)	Ib	50	Avelumab	2nd	6	2.6	10.6
Carneiro *et al.* (2019)	II	10	Nivolumab	≥2	10	1.8	N/A
Habra *et al.* (2019)	II, 1 site	16	Pembrolizumab	2nd	14	N/A	N/A
Head *et al.* (2019)	Retrospective	6	Pembrolizumab + mitotane	≥2	33	N/A	N/A
Raj *et al.* (2020)	II, 1 site	39	Pembrolizumab	Any	23	2.1	24.9
Remde *et al.* (2023)	Retrospective	54	ICI	≥2	13.5	3.0	10.4
**Trials with dual immunotherapy**							
Klein *et al.* (2021)	II	6	Nivolumab + ipilimumab	Any	33	N/A	N/A
McGregor *et al.* (2021)	II	18	Nivolumab + ipilimumab	Any	6	4.5	N/A
Baudin *et al.* (2022)	I/II	38	EO2401 + nivolumab	Any	12	1.9	N/A
**Trials with ICI and targeted therapy**							
Bedrose *et al.* (2020)	Retrospective	8	Pembrolizumab + lenvatinib	2nd or 3rd	25	5.5	N/A
Zhu *et al.* (2024)	II	21	Camrelizumab + apatinib	≥2	52	12.6	20.6
Grande *et al.* (2024)	II	24	Atezolizumab + cabozantinib	≥2	8.3	2.9	13.5

Abbreviations: ICI, immune checkpoint inhibitors; N, number of patients included; ORR, objective response rate; PFS, progression-free survival; OS, overall survival; 2nd, second; 3rd, third.

## Targeted therapies and future view

Key pathways involved in ACC pathogenesis that could be potentially targetable are in development. However, results from targeted therapies remain modest, highlighting the unmet need for novel treatments or effective drug combinations.

### IGF pathway

The IGF1R pathway has been extensively studied, but clinical trials have shown limited efficacy. Linsitinib, an IGF1R and insulin receptor (IR) antagonist, demonstrated two partial responses in a phase I trial with 15 ACC patients (Lee *et al.* 2020). However, the phase III GALACCTIC trial comparing linsitinib to placebo in 90 patients showed no significant differences in OS or PFS in second line (Fassnacht *et al.* 2015). Cixutumumab, a monoclonal antibody blocking IGF1R, was investigated with temsirolimus (mTOR inhibitor) in a trial with 26 heavily pretreated patients, resulting in no PRs but stabilization of disease (SD) in 11 patients for over 6 months (Naing *et al.* 2013). Another trial of cixutumumab monotherapy showed no responses or stabilization (Weigel *et al.* 2014). A phase II trial of cixutumumab with mitotane included 20 first-line patients and reported an ORR of 5% and a PFS of 6 weeks, which is unfavorable compared to the EDP-M regimen (20 weeks) (Lerario *et al.* 2014). Figitumumab, another monoclonal IGF1R antibody, also yielded poor results (Haluska *et al.* 2010). New strategies to enhance IGF1R antibody efficacy include antibody–drug conjugates and radiolabeling with α and β emitters (Solomon *et al.* 2019, 2020).

mTOR inhibition represents another potential therapeutic target within the IGF1 pathway; however, few ACC patients have been included in trials, with no noted responses (Ganesan *et al.* 2013, Wagle *et al.* 2014). A preclinical study using the H295R ACC cell model found that metformin significantly reduced cell viability and proliferation in a dose- and time-dependent manner. This effect was associated with the inhibition of ERK1/2 and mTOR phosphorylation and stimulation of AMPK activity. Metformin-treated cells exhibited lower levels of anti-apoptotic proteins Bcl-2 and Bcl-w, uncleaved caspase 3 and heat shock proteins HSP27, HSP60 and HSP70. In addition, metformin interferes with the IGF2/IGF-1R autocrine loop, which supports adrenal cancer growth. However, no combinations of treatments with metformin have been tested in ACC patients.

Inhibition of peroxisome proliferator-activated receptor (PPAR)-γ, a downstream effector of the IGF1R pathway, is another potential therapeutic target for ACC. Rosiglitazone, the thiazolidinedione with the highest affinity for PPAR-γ, has been shown to induce apoptosis in ACC cell lines and xenograft models while reducing VEGF expression and the anti-apoptotic marker Bcl-2 (Betz *et al.* 2005, Luconi *et al.* 2010).

### Tyrosine kinase inhibitors

EGFR is strongly expressed in 36% of ACC samples, leading to trials with anti-EGFR molecules. Gefitinib was the first EGFR inhibitor tested in ACC patients who had progressed on standard treatments, but no responses were reported (Samnotra *et al.* 2007). A combination of erlotinib and gemcitabine was evaluated in ten ACC patients who had undergone multiple cytotoxic therapies, revealing only one minor partial response lasting 8 months (Quinkler *et al.* 2008).

Derazantinib, an FGFR inhibitor, was tested in a basket phase II trial that included four ACC patients. One patient with FGFR1 amplification achieved a 20% tumor reduction, maintained for 3.5 years, while another patient with no detectable FGFR alterations had disease stabilization for over 12 months (Papadopoulos *et al.* 2017).

Broad-spectrum TKIs targeting VEGF receptors and other receptors have shown mixed results in ACC (Esteban-Villarrubia *et al.* 2020). Bevacizumab, combined with capecitabine in ten refractory ACC patients, yielded no objective responses or stabilization (Wortmann *et al.* 2010). Sunitinib was tried in a phase II study with 39 patients, with no observed responses and a PFS of 83 days; only five patients had disease control for at least 12 weeks (Kroiss *et al.* 2011, 2012). Axitinib was investigated alone in 13 patients without any responses (O’Sullivan *et al.* 2014). A phase II trial of dovitinib in 17 patients treated only with mitotane showed one partial response and 23% stabilization for at least 6 months, but the primary endpoint was not met (García-Donas *et al.* 2014). Cabozantinib was studied in a single-arm phase II trial in 18 patients with ACC, with an ORR of 11% and a PFS of 6 months (Campbell *et al.* 2024). The main IGF1R and TKI clinical trials are listed in Table 9.

**Table 9:** Completed phase 2/3 trials in ACC with I IGF1R antagonist and tyrosine kinase inhibitors.

Phase	NCT	Patients	Drugs	ORR	PFS	OS
III	NCT00924989 (Fassnacht *et al.* 2015)	139	Linsitinib vs placebo	3%	1.46 vs 1.53 m	10.76 days vs 11.86 m
II	NCT00831844 (Weigel *et al.* 2014)	10	Cixutumumab	0%	NR	NR
II	NCT00778817 (Naing *et al.* 2013)	20	Cixutumumab + mitotane	5%	1.4 m	NR
II	NCT00215202 (Samnotra *et al.* 2007)	19	Gefitinib	0%	NR	NR
II	NCT00453895 (Kroiss *et al.* 2012)	35	Sunitinib ± mitotane	0%	2.76 m	5.4 m
II	NCT01255137 (O’Sullivan *et al.* 2014)	13	Axitinib	0%	5.48 m	13.7 m
II	NCT01514526 (García-Donas *et al.* 2014)	17	Dovitinib	5%	1.8 m	NR
II	NCT 03370718 (Campbell *et al.* 2024)	18	Cabozantinib	11%	6 m	24 m

Abbreviations: NCT, National Clinical Trials Number; ORR, overall response rate; PFS, progression-free survival; OS, overall survival; NR, not reported; m, months.

### Future potential new targets

Some molecular pathways involved in ACC may represent potential therapeutic targets, although the low incidence and lack of general knowledge about this disease limit their development.

Inhibiting the Wnt/β-catenin pathway is a potential therapeutic target due to its role in some ACC patients. However, this pathway is ubiquitous, and its inhibition could lead to unknown adverse effects. PNU-74654 (Leal *et al.* 2015), rottlerin (Zhu *et al.* 2017), CWP291 (Lee *et al.* 2020), tegavivint (Cranmer *et al.* 2022) and porcupine inhibitors have been tested in early phase I studies, although not specifically in ACC (Koo *et al.* 2015).

p53/Rb pathway plays a central role in ACC pathogenesis, with frequent mutations (*TP53*, *CDKN2A*, *CDK4*, *CDK6*, *MDM2* and *RB1*) making it a key target for ACC treatment. CDK4/6 inhibitors (palbociclib, ribociclib and abemaciclib) represent potential treatments; although no ACC-specific CDK inhibitor trials exist yet. Palbociclib has shown significant cell viability reduction and cell cycle arrest *in vitro*, effects enhanced by combining it with linsitinib (IGF inhibitor) (Liang *et al.* 2020). Other p53/Rb pathway-related drugs, such as *MDM2* inhibitors or mutant *p53* inhibitors, are in development (Konopleva *et al.* 2020, Wang *et al.* 2023).

Another potential target of future treatments is the inhibition of delta-like noncanonical Notch ligand 1 (DLK1) that is highly expressed in ACC, and growing evidence suggest that DLK1 expression in cancer is associated with worse prognosis and that DLK1 may be a marker of cancer stem cells (Pittaway *et al.* 2021). An ongoing clinical trial (NCT06041516) is focused on testing the efficacy of an antibody–drug conjugate ADCT-701, which is a humanized antibody directed against *DLK1*.

## Prognosis and follow-up

### Prognosis

The prognosis for ACC is generally poor but highly heterogeneous, underscoring the need for reliable prognostic tools to guide follow-up, adjuvant treatment and patient counseling (Elhassan *et al.* 2021). Prognostic classifications fall into two categories: clinical and molecular.

The clinical classification is based on the TNM-ENSAT staging system, which stratifies patients by tumor extent and guides treatment decisions (Fassnacht *et al.* 2018). Tumor stage at diagnosis is the strongest predictor of outcome, with metastases indicating the worst prognosis. The ENSAT system relies on comprehensive preoperative imaging, systematic lymph node resection and detailed surgical and pathological reports (Fassnacht *et al.* 2018).

A modified ENSAT (mENSAT) stage has been proposed for advanced disease, incorporating nodal involvement (equivalent to stage IV) and the number of metastatic organs: IVa (two organs), IVb (three organs) and IVc (>3 organs) (Libé *et al.* 2015) (Table 10). The **GRAS criteria** (grade, resection status, age and secretory syndrome) provide an independent framework for predicting relapse and survival across all stages (Elhassan *et al.* 2021). GRAS factors include grade (Weiss >6 and/or Ki67 ≥ 20%), resection margin status (R0-R2), age (< or ≥50 years) and cortisol excess (S). Elhassan *et al.* (2021), introduced the S-GRAS score, which assigns points to these variables, and demonstrated superior prognostic accuracy compared to ENSAT staging and Ki67 index alone (Beuschlein *et al.* 2015). In a multicenter study of 942 ACC patients, 5-year survival decreased from 74% (S-GRAS score 0) to 9% (score 6).

**Table 10:** Proposed modified ENSAT stages. Based on Fassnacht *et al.* (2009) and Libé *et al.* 2015.

TNM	mENSAT	
T1: Tumor located in the adrenal gland ≤5 cm T2: tumor located in the adrenal gland >5 cm T3: tumor infiltrating adipose tissue T4: tumor invasion into adjacent organs or venous tumor thrombus in vena cava or renal vein N0: no positive lymph node N1: positive lymph node M0: no distant metastases M1: presence of distant metastases	I II III IV	T1 N0 M0 T2 N0 M0 T3 or T4 N0 M0 Any T, N1 M1: IVa: 2 organs IVb: 3 organs IVc: >3 organs

T, tumor; N, lymph nodes; M, metastases; mENSAT, modified ENSAT staging.

Molecular analysis is not routinely performed but holds promise for future prognostic tools. In contrast, the S-GRAS score, already part of standard clinical evaluation for surgical patients, requires prospective validation to confirm its role in predicting recurrence and response to mitotane. Future studies should also investigate the integration of GRAS components into mENSAT classification for non-resectable ACC (Elhassan *et al.* 2021).

### Follow-up

**Figure 6:**
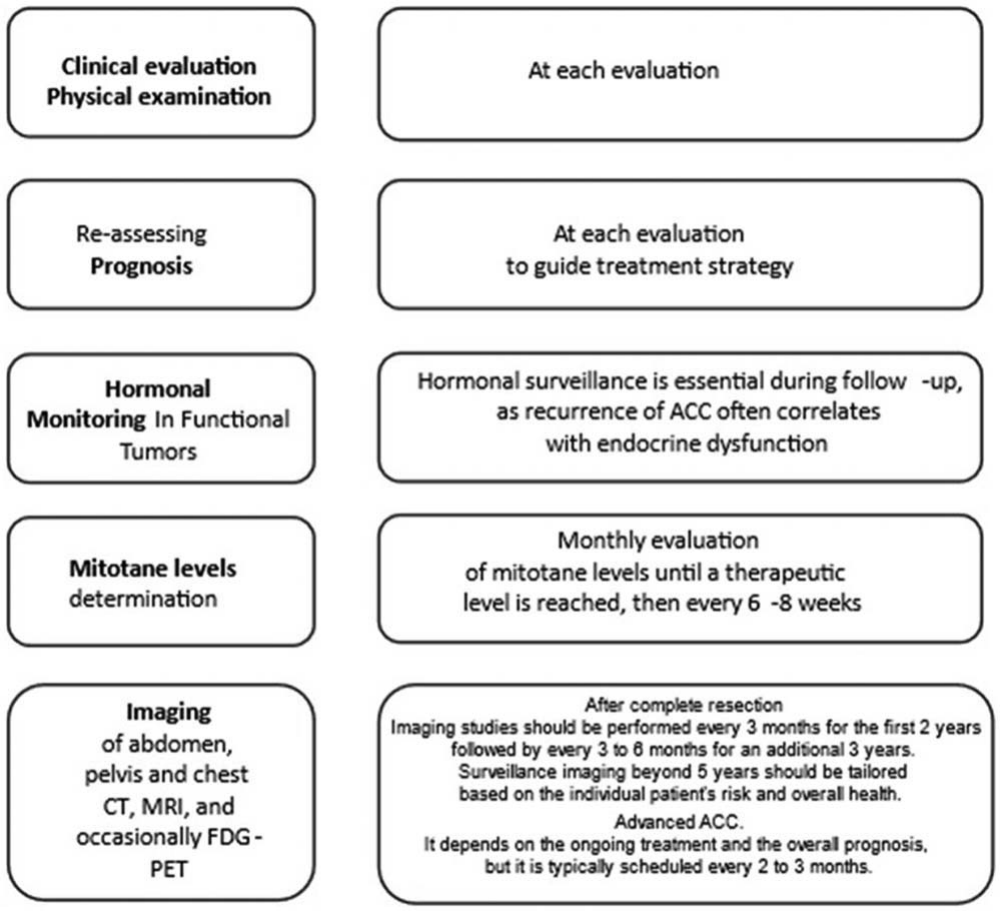
Recommendations for ACC follow-up. Based on Fassnacht *et al.* (2010), (2018), Gaujoux & Brennan (2012). Abbreviations: CT, computed tomography; MRI, magnetic resonance imaging; FDG PET, fluorodeoxyglucose positron-emission tomography/computed tomography.

Surveillance strategies for ACC remain underexplored (Fassnacht *et al.* 2010). However, due to its high recurrence rate even after successful treatment, rigorous follow-up is necessary for detecting recurrences, metastases, endocrine dysfunction and evaluating treatment response. A comprehensive approach that includes clinical assessment, imaging and hormonal monitoring is essential (Fig. 6).

Postoperative follow-up typically involves regular imaging, clinical assessments and laboratory tests. The ENSAT recommends imaging studies, such as CT or MRI, to monitor local recurrence or distant metastases, particularly during the first 3–5 years post-surgery, when the risk of recurrence is highest. Imaging frequency is more intense in the first 2 years, with intervals of 3–6 months, becoming less frequent after 5 years if the patient remains disease-free (Fassnacht *et al.* 2018). The S-GRAS score may help determine follow-up intervals (Elhassan *et al.* 2021).

## Special situations in patients with ACC

### Pregnancy and ACC

The management of ACC during pregnancy remains challenging due to its rarity and limited evidence. Early, individualized and multidisciplinary treatment involving endocrinology, surgery, obstetrics and psychology is essential (Fassnacht *et al.* 2018).

A retrospective study of 110 female ACC patients showed that tumors diagnosed during pregnancy or postpartum are more likely to be hormone-secreting and advanced stages, resulting in poorer OS and higher fetal morbidity and mortality compared to non-pregnant women (Abiven-Lepage *et al.* 2010). ACC in pregnancy is often linked to hypercortisolism, which increases maternal and fetal risks (Puglisi *et al.* 2023*a*) Clinical features of Cushing’s syndrome (CS) often overlap with normal pregnancy, delaying diagnosis. Reliable diagnostic indicators include a more than threefold increase in 24hUFC and elevated nighttime salivary cortisol levels (Hamblin *et al.* 2022, Morris *et al.* 2023, Stoinis *et al.* 2024).

Pregnancy may promote aggressive ACC independently of CS (Abiven-Lepage *et al.* 2010, Fassnacht *et al.* 2018). Molecular features, such as elevated IGF2, increased progesterone and estrogen receptor expression and low CREB expression, suggest a unique molecular pattern in pregnancy-associated ACC, although further studies are needed (Faillot & Assie 2016).

Complete tumor resection via adrenal surgery is recommended, regardless of gestational age, following confirmation by MRI. Collaboration with an obstetric team is essential due to increased risk of preterm delivery, particularly in the third trimester.

In first trimester stages III or IV, abortion may need to be discussed. Mitotane is contraindicated during pregnancy due to teratogenic and adrenolytic effects (Baszko-Błaszyk *et al.* 2011, Tripto-Shkolnik *et al.* 2013), although metyrapone and ketoconazole have been used to control cortisol secretion. Postpartum, aggressive treatment is advised, even after apparent complete surgical resection. Adjuvant mitotane should begin as soon as possible, and breastfeeding is not recommended (Abiven-Lepage *et al.* 2010, Fassnacht *et al.* 2018).

Women on mitotane require effective contraception to prevent pregnancy, as teratogenic effects persist until drug levels are undetectable, a process that may exceed 6 months. Non-estrogenic contraceptives are preferred due to ACC’s potential interaction with estrogen pathways. Pregnancy after successful ACC treatment does not appear to worsen clinical outcomes, but patients should be counseled on the significant risk of recurrence in the first few years post-diagnosis (De Corbière *et al.* 2015, Fassnacht *et al.* 2018, Szkodziak *et al.* 2024).

### Hormone-producing ACC

ACC can lead to adrenal hormone overproduction, causing rapid and severe clinical symptoms (Table 1) (Fassnacht *et al.* 2018). Hormonal phenotypes include cortisol excess (CS), androgen overproduction and less commonly, mineralocorticoid or estradiol secretion. Hormonal assessment, as outlined in the diagnostic section, is critical for identifying secretory profiles and guiding management.

Up to 50–70% of ACCs are hormonally active, the most frequent cortisol. Symptoms include myopathy, hypokalemia, wasting, weight loss, hyperglycemia, osteoporosis facial plethora and edemas. Androgen secretion (20–30%) causes virilization, hirsutism, alopecia and menstrual alterations in women, while estrogen secretion (5% of males) results in painful gynecomastia and testicular atrophy. Mineralocorticoid hypersecretion, with the appearance of hypertension and hypokalemia, is rare (2–3% of ACC) and often due to precursors such as 11-deoxycorticosterone (Berruti *et al.* 2014, Fassnacht *et al.* 2018).

Despite inefficient hormone production in some ACCs, elevated metabolites, such as 5-pregnanetriol and tetrahydro-11-deoxycortisol can aid in diagnosis and monitoring. Mass spectrometry offers a promising tool for assessing recurrence and treatment response (Arlt *et al.* 2011, Chortis *et al.* 2020, Gadelha *et al.* 2023, Kimpel *et al.* 2023, Vogg *et al.* 2023).

Managing hormone excess requires a multidisciplinary approach tailored to the severity of and symptoms and comorbidities (Fassnacht *et al.* 2018). Intraoperative and postoperative glucocorticoid replacement therapy, preferably with hydrocortisone, is indicated in all patients, with evidence of possible autonomous cortisol secretion with cortisol post-dexamethasone suppression test >1.8 μg/dL (50 nmol/L). This should follow the suggestions for major stress dose replacement as per recent international guidelines (Araujo Castro *et al.* 2019, Castinetti *et al.* 2021). Postoperatively, the dose of glucocorticoid should be tapered on an individualized basis by a physician experienced.

**Mitotane** is effective for mild hormone excess but requires weeks to achieve therapeutic levels, necessitating faster-acting agents in severe Cushing syndrome (Supplementary Material S2) (Fassnacht *et al.* 2018).

- **First-line agents:** metyrapone (CYP11B1 inhibitor) is well-tolerated, even with mitotane or chemotherapy. Osilodrostat inhibits cortisol and aldosterone synthesis, offering rapid control of cortisol levels with fewer androgenic adverse effects (Bonnet-Serrano *et al.* 2022, Detomas *et al.* 2022, Tabarin *et al.* 2022, Capatina *et al.* 2024).
- **Alternative therapies:** ketoconazole inhibits multiple steroidogenesis steps but is less effective, interacts with mitotane (via CYP3A4/P450 induction) and has hepatotoxicity risks. Levoketoconazole and etomidate are emerging options for selected patients (Fassnacht *et al.* 2018, Castinetti *et al.* 2021, Capatina *et al.* 2024). Etomidate is the only intravenous treatment available for Cushing syndrome
- **Receptor antagonists:** mifepristone and relacorilant (selective glucocorticoid receptor antagonist) are used in CS with increased hypertension and hypokalemia.
- **Mineralocorticoid receptor antagonists (spironolactone and eplerenone):** used for specific complications in CS, such as hypertension or hypokalemia (Fassnacht *et al.* 2018, Castinetti *et al.* 2021, Capatina *et al.* 2024).

Doses should be titrated to normalize hormone levels, or in the case of receptor antagonists to improved comorbidities, accepting that assessment of this can be challenging in cancer patients. Block-and-replace therapies are valuable in concomitant treatment with mitotane to avoid acute adrenal insufficiency. Hormonal substitutive therapy and evaluation of the adverse effects must be adapted considering all concomitant therapies and specific CYP-P450 interferences, action on CBG, thyroid and metabolic function (Fassnacht *et al.* 2018, Castinetti *et al.* 2021, Capatina *et al.* 2024).

All patients treated with enzyme inhibitors or receptor antagonists need to be educated about symptoms and signs of adrenal insufficiency (Neumann *et al.* 2019). All patients at risk for adrenal insufficiency need to be supplied with emergency medication and instructions (Araujo Castro *et al.* 2019). Glucocorticoid withdrawal syndrome should be taken in account, although diagnosis is difficult (Zhang & Ioachimescu 2024).

Prophylaxis against thromboembolism and infections (e.g., pneumocystis pneumonia) is essential in hypercortisolemic patients. Electrolyte imbalances, including hypokalemia, should be corrected with potassium supplements and monitoring at least once a week (Puglisi *et al.* 2018*b*, Fallo *et al.* 2022, Mehlich *et al.* 2023, Capatina *et al.* 2024).

Androgen secretion in women can affect the quality of life, leading to hirsutism and virilization. Treatment options include androgen receptor antagonists such as bicalutamide, flutamide or spironolactone (Fassnacht *et al.* 2018). Aldosterone-producing tumors are less frequent leading to hypertension and/or hypokalemia. High doses of mineralocorticoid receptor antagonists such as spironolactone and eplerenone are indicated (Fassnacht *et al.* 2018). Estradiol-producing ACC in male patients can be treated with estrogen receptor antagonists or aromatase inhibitors (Fassnacht *et al.* 2018).

## Conclusions

ACC is a rare endocrine malignancy, with a poor overall prognosis reflected in a 5-year survival rate of approximately 35%. Surgery remains the only curative option when complete resection is feasible. For unresectable or metastatic ACC, current therapeutic standards include mitotane, chemotherapy, radiotherapy and locoregional treatments. Despite advances in understanding ACC pathogenesis, outcomes with emerging therapies, including immunotherapy, tyrosine kinase inhibitors and other targeted agents, are still modest. These findings underscore the need for more studies with innovative treatments and novel combinations of existing drugs. Effective management of ACC requires a multidisciplinary team of specialists, including endocrinologists, surgeons and/or urologists, medical and radiation oncologists, pathologists and nuclear medicine physicians. This collaborative approach is crucial to improve treatment outcomes and care for patients with ACC.

## Supplementary materials



## Declaration of interest

MAC received speakers’ honoraria and consulting fees from Esteve and Recordati Rare Diseases. ACB received payment or honoraria for lectures, presentations, speakers, bureaus, manuscript writing, educational events or participation on a data safety monitoring board or advisory board from Astellas, AstraZeneca, BMS, Lilly, MSD, Ipsen, Novartis and Esteve. PJF received honoraria for speakers' bureau participation, and serving on advisory boards from Astellas, AstraZeneca, Bristol-Myers Squibb (BMS), Esteve, Merck Sharp & Dohme (MSD), Novartis, Nutricia, Pfizer, Rovi, Takeda and Viatris and research grants from Astellas, AstraZeneca, BMS and MSD. MRF received honoraria for lectures, manuscript writings, educational events or advisory boards from Telix, Sirtex Medical , Janssen, Novartis and Astellas. FAH received honoraria for lectures and advisor board from HRA Pharma and Recordatti. JLVC received honoraria for lectures, traveling, educational events or advisory boards from Novartis, Sirtex Medical and Terumo. The authors declare no potential conflict of interest.

## Funding

This work was funded by Sociedad Española de Endocrinología y Nutrición (SEEN) and the Spanish Group of Neuroendocrine and Endocrine Tumors (GETNE).

## References

[bib1] Abiven-Lepage G, Coste J, Tissier F, et al. 2010 Adrenocortical carcinoma and pregnancy: clinical and biological features and prognosis. Eur J Endocrinol 163 793–800. (10.1530/EJE-10-0412) 20699382

[bib2] Abiven G, Coste J, Groussin L, et al. 2006 Clinical and biological features in the prognosis of adrenocortical cancer: poor outcome of cortisol-secreting tumors in a series of 202 consecutive patients. J Clin Endocrinol Metab 91 2650–2655. (10.1210/JC.2005-2730) 16670169

[bib3] Abraham J, Bakke S, Rutt A, et al. 2002 A phase II trial of combination chemotherapy and surgical resection for the treatment of metastatic adrenocortical carcinoma: continuous infusion doxorubicin, vincristine, and etoposide with daily mitotane as a P-glycoprotein antagonist. Cancer 94 2333–2343. (10.1002/CNCR.10487) 12015757

[bib4] Ahmed AA, Thomas AJ, Ganeshan DM, et al. 2020 Adrenal cortical carcinoma: pathology, genomics, prognosis, imaging features, and mimics with impact on management. Abdom Radiol 45 945–963. (10.1007/S00261-019-02371-Y) 31894378

[bib5] Al Asadi A, Hubbs DM, Sweigert PJ, et al. 2021 Analysis of adjuvant chemotherapy in patients undergoing curative-intent resection of localized adrenocortical carcinoma. Am J Surg 222 119–125. (10.1016/J.AMJSURG.2020.10.038) 33168156

[bib6] Allolio B & Fassnacht M 2006 Clinical review: adrenocortical carcinoma: clinical update. J Clin Endocrinol Metab 91 2027–2037. (10.1210/JC.2005-2639) 16551738

[bib7] Anderson KL, Adam MA, Thomas SM, et al. 2018 Impact of micro- and macroscopically positive surgical margins on survival after resection of adrenocortical carcinoma. Ann Surg Oncol 25 1425–1431. (10.1245/S10434-018-6398-5) 29500765

[bib8] Araujo Castro M, Currás Freixes M, de Miguel Novoa P, et al. 2019 SEEN guidelines for the management and prevention of acute adrenal insufficiency. Endocrinologia, Diabetes y Nutricion 67 53–60. (10.1016/j.endinu.2019.01.004) 31003863

[bib9] Araujo-Castro M, Iturregui Guevara M, Calatayud Gutiérrez M, et al. 2020 Practical guide on the initial evaluation, follow-up, and treatment of adrenal incidentalomas Adrenal Diseases Group of the Spanish Society of Endocrinology and Nutrition. Endocrinol Diabetes Nutr 67 408–419. (10.1016/j.endinu.2020.03.002) 32349941

[bib10] Araujo-Castro M, Pascual-Corrales E, Molina-Cerrillo J, et al. 2021 Immunotherapy in adrenocortical carcinoma: predictors of response, efficacy, safety, and mechanisms of resistance. Biomedicines 9 304. (10.3390/BIOMEDICINES9030304) 33809752 PMC8002272

[bib11] Arlt W, Biehl M, Taylor AE, et al. 2011 Urine steroid metabolomics as a biomarker tool for detecting malignancy in adrenal tumors. J Clin Endocrinol Metab 96 3775–3784. (10.1210/jc.2011-1565) 21917861 PMC3232629

[bib12] Assié G, Letouzé E, Fassnacht M, et al. 2014 Integrated genomic characterization of adrenocortical carcinoma. Nat Genet 46 607–612. (10.1038/ng.2953) 24747642

[bib13] Basile V, Puglisi S, Altieri B, et al. 2021 What is the optimal duration of adjuvant mitotane therapy in adrenocortical carcinoma? An unanswered question. J Personalized Med 11 269. (10.3390/JPM11040269) PMC8066814 33916613

[bib14] Baszko-Błaszyk D, Ochmańska K, Waśko R, et al. 2011 Pregnancy in a patient with adrenocortical carcinoma during treatment with mitotane – a case report. Endokrynol Pol 62 186–188. (https://pubmed.ncbi.nlm.nih.gov/21528483/) 21528483

[bib15] Baudin E, Docao C, Gicquel C, et al. 2002 Use of a topoisomerase I inhibitor (irinotecan, CPT-11) in metastatic adrenocortical carcinoma. Ann Oncol 13 1806–1809. (10.1093/ANNONC/MDF291) 12419755

[bib16] Baudin E, Jimenez C, Fassnacht M, et al. 2022 EO2401, a novel microbiome-derived therapeutic vaccine for patients with adrenocortical carcinoma (ACC): preliminary results of the SPENCER study. J Clin Oncol 40 (Supplement 16) 4596. (10.1200/JCO.2022.40.16_SUPPL.4596)

[bib17] Bechmann N, Moskopp ML, Ullrich M, et al. 2020 HIF2α supports pro-metastatic behavior in pheochromocytomas/paragangliomas. Endocr Relat Cancer 27 625–640. (10.1530/ERC-20-0205) 33112842

[bib18] Bedrose S, Miller KC, Altameemi L, et al. 2020 Combined lenvatinib and pembrolizumab as salvage therapy in advanced adrenal cortical carcinoma. J ImmunoTherapy Cancer 8 e001009. (10.1136/jitc-2020-001009) PMC7394183 32737143

[bib19] Berruti A, Fassnacht M, Haak H, et al. 2014 Prognostic role of overt hypercortisolism in completely operated patients with adrenocortical cancer. Eur Urol 65 832–838. (10.1016/J.EURURO.2013.11.006) 24268504

[bib20] Betz MJ, Shapiro I, Fassnacht M, et al. 2005 Peroxisome proliferator-activated receptor-gamma agonists suppress adrenocortical tumor cell proliferation and induce differentiation. J Clin Endocrinol Metab 90 3886–3896. (10.1210/JC.2004-1267) 15886257

[bib21] Beuschlein F, Weigel J, Saeger W, et al. 2015 Major prognostic role of Ki67 in localized adrenocortical carcinoma after complete resection. J Clin Endocrinol Metab 100 841–849. (10.1210/JC.2014-3182) 25559399

[bib22] Bilimoria KY, Shen WT, Elaraj D, et al. 2008 Adrenocortical carcinoma in the United States: treatment utilization and prognostic factors. Cancer 113 3130–3136. (10.1002/CNCR.23886) 18973179

[bib23] Bisceglia M, Ludovico O, Di Mattia A, et al. 2004 Adrenocortical oncocytic tumors: report of 10 cases and review of the literature. Int J Surg Pathol 12 231–243. (10.1177/106689690401200304) 15306935

[bib24] Boilève A, Mathy E, Roux C, et al. 2021 Combination of mitotane and locoregional treatments in low-volume metastatic adrenocortical carcinoma. J Clin Endocrinol Metab 106 E4698–E4707. (10.1210/clinem/dgab449) 34143888

[bib25] Bonnet-Serrano F, Poirier J, Vaczlavik A, et al. 2022 Differences in the spectrum of steroidogenic enzyme inhibition between Osilodrostat and Metyrapone in ACTH-dependent Cushing syndrome patients. Eur J Endocrinol 187 315–322. (10.1530/EJE-22-0208) 35699971

[bib26] Borges KS, Pignatti E, Leng S, et al. 2020 Wnt/β-catenin activation cooperates with loss of p53 to cause adrenocortical carcinoma in mice. Oncogene 39 5282–5291. (10.1038/S41388-020-1358-5) 32561853 PMC7378041

[bib27] Bougeard G, Sesboue R, Baert-Desurmont S, et al. 2008 Molecular basis of the Li-Fraumeni syndrome: an update from the French LFS families. J Med Genet 45 535–538. (10.1136/jmg.2008.057570) 18511570

[bib28] Byung KP, Chan KK, Kim B, et al. 2007 Comparison of delayed enhanced CT and chemical shift MR for evaluating hyperattenuating incidental adrenal masses. Radiology 243 760–765. (10.1148/RADIOL.2433051978) 17517932

[bib29] Calabrese A, Basile V, Puglisi S, et al. 2019 Adjuvant mitotane therapy is beneficial in non-metastatic adrenocortical carcinoma at high risk of recurrence. Eur J Endocrinol 180 387–396. (10.1530/EJE-18-0923) 30991359

[bib30] Calabrese A, Puglisi S, Borin C, et al. 2023 The management of postoperative disease recurrence in patients with adrenocortical carcinoma: a retrospective study in 106 patients. Eur J Endocrinol 188 118–124. (10.1093/EJENDO/LVAD002) 36655273

[bib31] Campbell MT, Balderrama-Brondani V, Jimenez C, et al. 2024 Cabozantinib monotherapy for advanced adrenocortical carcinoma: a single-arm, phase 2 trial. Lancet Oncol 25 649–657. (10.1016/S1470-2045(24)00095-0) 38608694

[bib32] Capatina C, Hanzu FA, Hinojosa-Amaya JM, et al. 2024 Medical treatment of functional pituitary adenomas, trials and tribulations. J Neuro Oncol 168 197–213. (10.1007/S11060-024-04670-X) 38760632

[bib33] Carmona-Bayonas A, Álvarez-Escolá C, Ballester Navarro I, et al. 2025 Does adjuvant mitotane impact cure rates in adrenocortical carcinoma? Insights from the ICARO-GETTHI/SEEN registry. J Clin Endocrinol Metab dgaf082. (10.1210/clinem/dgaf082) 39988973

[bib34] Carneiro BA, Konda B, Costa RB, et al. 2019 Nivolumab in metastatic adrenocortical carcinoma: results of a phase 2 trial. J Clin Endocrinol Metab 104 6193–6200. (10.1210/jc.2019-00600) 31276163

[bib35] Castinetti F, Nieman LK, Reincke M, et al. 2021 Approach to the patient treated with steroidogenesis inhibitors. J Clin Endocrinol Metab 106 2114–2123. (10.1210/CLINEM/DGAB122) 33675650 PMC8427736

[bib36] Cazejust J, De Baère T, Auperin A, et al. 2010 Transcatheter arterial chemoembolization for liver metastases in patients with adrenocortical carcinoma. J Vasc Interv Radiol 21 1527–1532. (10.1016/J.JVIR.2010.05.020) 20801688

[bib37] Chagpar R, Siperstein AE & Berber E 2014 Adrenocortical cancer update. Surg Clin 94 669–687. (10.1016/J.SUC.2014.02.009) 24857583

[bib38] Chopra S, Walia R, Mathur Y, et al. 2023 68 Ga-DOTA.SA.FAPI as a potential, noninvasive diagnostic probe for recurrent and metastatic adrenocortical carcinoma: a head-to-head comparison with 18F-FDG. Clin Nucl Med 48 E173–E175. (10.1097/RLU.0000000000004563) 36727882

[bib39] Chortis V, Taylor AE, Schneider P, et al. 2013a Mitotane therapy in adrenocortical cancer induces CYP3A4 and inhibits 5α-reductase, explaining the need for personalized glucocorticoid and androgen replacement. J Clin Endocrinol Metab 98 161–171. (10.1210/JC.2012-2851) 23162091

[bib40] Chortis V, Taylor AE, Schneider P, et al. 2013b Mitotane therapy in adrenocortical cancer induces CYP3A4 and inhibits 5α-reductase, explaining the need for personalized glucocorticoid and androgen replacement. J Clin Endocrinol Metab 98 161–171. (10.1210/jc.2012-2851) 23162091

[bib41] Chortis V, Bancos I, Nijman T, et al. 2020 Urine steroid metabolomics as a novel tool for detection of recurrent adrenocortical carcinoma. J Clin Endocrinol Metab 105 e307–e318. (10.1210/clinem/dgz141) 31665449 PMC7112967

[bib42] Cooper AB, Habra MA, Grubbs EG, et al. 2013 Does laparoscopic adrenalectomy jeopardize oncologic outcomes for patients with adrenocortical carcinoma? Surg Endosc 27 4026–4032. (10.1007/S00464-013-3034-0) 23765427

[bib43] Cosentini D, Badalamenti G, Grisanti S, et al. 2019 Activity and safety of temozolomide in advanced adrenocortical carcinoma patients. Eur J Endocrinol 181 681–689. (10.1530/EJE-19-0570) 31639772

[bib44] Cranmer LD, Razak ARA, Ratan R, et al. 2022 Results of a phase I dose escalation and expansion study of tegavivint (BC2059), a first-in-class TBL1 inhibitor for patients with progressive, unresectable desmoid tumor. J Clin Oncol 40 (Supplement 16) 11523. (10.1200/JCO.2022.40.16_SUPPL.11523)

[bib45] Datta J & Roses RE 2016 Surgical management of adrenocortical carcinoma: an evidence-based approach. Surg Oncol Clin N Am 25 153–170. (10.1016/J.SOC.2015.08.011) 26610780

[bib46] de Corbière P, Ritzel K, Cazabat L, et al. 2015 Pregnancy in women previously treated for an adrenocortical carcinoma. J Clin Endocrinol Metab 100 4604–4611. (10.1210/JC.2015-2341) 26461265

[bib47] De Filpo G, Mannelli M & Canu L 2021 Adrenocortical carcinoma: current treatment options. Curr Opin Oncol 33 16–22. (10.1097/CCO.0000000000000695) 33186181

[bib48] de Ponthaud C, Bekada S, Buffet C, et al. 2024 Which lymphadenectomy for adrenocortical carcinoma? Surgery 176 1635–1644. (10.1016/J.SURG.2024.09.008) 39370320

[bib49] Del Rivero J, Else T, Hallanger-Johnson J, et al. 2024 A review of mitotane in the management of adrenocortical cancer. Oncologist 29 747–760. (10.1093/ONCOLO/OYAE084) 39037424 PMC11379655

[bib50] Detomas M, Altieri B, Deutschbein T, et al. 2022 Metyrapone versus osilodrostat in the short-term therapy of endogenous cushing’s syndrome: results from a single center cohort study. Front Endocrinol 13 903545. (10.3389/FENDO.2022.903545) PMC9235400 35769081

[bib51] Dickson PV, Kim L, Yen TWF, et al. 2018 Evaluation, staging, and surgical management for adrenocortical carcinoma: an update from the SSO endocrine and head and neck disease site working group. Ann Surg Oncol 25 3460–3468. (10.1245/S10434-018-6749-2) 30229419

[bib52] Donatini G, Caiazzo R, Do Cao C, et al. 2014 Long-term survival after adrenalectomy for stage I/II adrenocortical carcinoma (ACC): a retrospective comparative cohort study of laparoscopic versus open approach. Ann Surg Oncol 21 284–291. (10.1245/S10434-013-3164-6) 24046101

[bib53] Dreher N, Hahner S, Fuß CT, et al. 2024 CXCR4-directed PET/CT with [68 Ga]Ga-pentixafor in solid tumors-a comprehensive analysis of imaging findings and comparison with histopathology. Eur J Nucl Med Mol Imag 51 1383–1394. (10.1007/S00259-023-06547-Z) PMC10957681 38082196

[bib54] Elhassan YS, Altieri B, Berhane S, et al. 2022 S-GRAS score for prognostic classification of adrenocortical carcinoma: an international, multicenter ENSAT study. Eur J Endocrinol 186 25–36. (10.1530/EJE-21-0510) PMC8679848 34709200

[bib55] Else T, Williams AR, Sabolch A, et al. 2014 Adjuvant therapies and patient and tumor characteristics associated with survival of adult patients with adrenocortical carcinoma. J Clin Endocrinol Metab 99 455–461. (10.1210/JC.2013-2856) 24302750 PMC3913818

[bib56] Erdogan I, Deutschbein T, Jurowich C, et al. 2013 The role of surgery in the management of recurrent adrenocortical carcinoma. J Clin Endocrinol Metab 98 181–191. (10.1210/JC.2012-2559) 23150691

[bib57] Esteban-Villarrubia J, Soto-Castillo JJ, Pozas J, et al. 2020 Tyrosine kinase receptors in oncology. Int J Mol Sci 21 8529–8548. (10.3390/IJMS21228529) 33198314 PMC7696731

[bib58] Faillot S & Assie G 2016 The genomics of adrenocortical tumors. Eur J Endocrinol 174 R249–R265. (10.1530/EJE-15-1118) 26739091

[bib59] Fallo F, Di Dalmazi G, Beuschlein F, et al. 2022 Diagnosis and management of hypertension in patients with Cushing’s syndrome: a position statement and consensus of the Working Group on Endocrine Hypertension of the European Society of Hypertension. J Hypertens 40 2085–2101. (10.1097/HJH.0000000000003252) 35950979

[bib60] Fassnacht M, Hahner S, Polat B, et al. 2006 Efficacy of adjuvant radiotherapy of the tumor bed on local recurrence of adrenocortical carcinoma. J Clin Endocrinol Metab 91 4501–4504. (10.1210/JC.2006-1007) 16895957

[bib188] Fassnacht M, Johanssen S, Quinkler M, et al. 2009 Limited prognostic value of the 2004 International Union Against Cancer staging classification for adrenocortical carcinoma: proposal for a revised TNM classification. Cancer 115 243–250. (10.1002/cncr.24030) 19025987

[bib61] Fassnacht M, Johanssen S, Fenske W, et al. 2010 Improved survival in patients with stage II adrenocortical carcinoma followed up prospectively by specialized centers. J Clin Endocrinol Metab 95 4925–4932. (10.1210/JC.2010-0803) 20668036

[bib62] Fassnacht M, Libé R, Kroiss M, et al. 2011 Adrenocortical carcinoma: a clinician’s update. Nat Rev Endocrinol 7 323–335. (10.1038/NRENDO.2010.235) 21386792

[bib63] Fassnacht M, Terzolo M, Allolio B, et al. 2012 Combination chemotherapy in advanced adrenocortical carcinoma. N Engl J Med 366 2189–2197. (10.1056/nejmoa1200966) 22551107

[bib64] Fassnacht M, Berruti A, Baudin E, et al. 2015 Linsitinib (OSI-906) versus placebo for patients with locally advanced or metastatic adrenocortical carcinoma: a double-blind, randomised, phase 3 study. Lancet Oncol 16 426–435. (10.1016/S1470-2045(15)70081-1) 25795408

[bib65] Fassnacht M, Dekkers OM, Else T, et al. 2018 European Society of Endocrinology Clinical Practice Guidelines on the management of adrenocortical carcinoma in adults, in collaboration with the European Network for the Study of Adrenal Tumors. Eur J Endocrinol 179 G1–G46. (10.1530/EJE-18-0608) 30299884

[bib66] Fassnacht M, Assie G, Baudin E, et al. 2020a Adrenocortical carcinomas and malignant phaeochromocytomas: ESMO-EURACAN Clinical Practice Guidelines for diagnosis, treatment and follow-up. Ann Oncol 31 1476–1490. (10.1016/J.ANNONC.2020.08.2099) 32861807

[bib67] Fassnacht M, Assie G, Baudin E, et al. 2020b Adrenocortical carcinomas and malignant phaeochromocytomas: ESMO–EURACAN Clinical Practice Guidelines for diagnosis, treatment and follow-up. Ann Oncol 31 1476–1490. (10.1016/j.annonc.2020.08.2099) 32861807

[bib68] Fassnacht M, Assie G, Baudin E, et al. 2023a Corrigendum to “Adrenocortical carcinomas and malignant phaeochromocytomas: ESMO-EURACAN Clinical Practice Guidelines for diagnosis, treatment and follow-up”: [Annals of Oncology volume 31 (2020) 1476–1490]. Ann Oncol 34 631. (10.1016/J.ANNONC.2022.12.006) 32861807

[bib69] Fassnacht M, Tsagarakis S, Terzolo M, et al. 2023b European Society of Endocrinology clinical practice guidelines on the management of adrenal incidentalomas, in collaboration with the European Network for the Study of Adrenal Tumors. Eur J Endocrinol 189 G1–G42. (10.1093/EJENDO/LVAD066) 37318239

[bib70] Fassnacht M, Puglisi S, Kimpel O, et al. 2025 Adrenocortical carcinoma: a practical guide for clinicians. Lancet Diabetes Endocrinol S2213-8587(24)00378-4. (10.1016/S2213-8587(24)00378-4) 40086465

[bib71] Fernandez Ranvier GG & Inabnet WB 2015 Surgical management of adrenocortical carcinoma. Endocrinol Metab Clin N Am 44 435–452. (10.1016/J.ECL.2015.02.008) 26038210

[bib72] Gadelha M, Gatto F, Wildemberg LE, et al. 2023 Cushing’s syndrome. Lancet 402 2237–2252. (10.1016/S0140-6736(23)01961-X) 37984386

[bib73] Ganesan P, Piha-Paul S, Naing A, et al. 2013 Phase I clinical trial of lenalidomide in combination with temsirolimus in patients with advanced cancer. Invest New Drugs 31 1505–1513. (10.1007/S10637-013-0013-1) 23982248

[bib74] García-Donas J, Hernando Polo S, Guix M, et al. 2014 Phase II study of dovitinib in first line metastatic or (non resectable primary) adrenocortical carcinoma (ACC): SOGUG study 2011-03. J Clin Oncol 32 (Supplement 15) 4588. (10.1200/JCO.2014.32.15_SUPPL.4588)

[bib75] Gaujoux S & Brennan MF 2012 Recommendation for standardized surgical management of primary adrenocortical carcinoma. Surgery 152 123–132. (10.1016/J.SURG.2011.09.030) 22306837

[bib76] Gaujoux S, Mihai R, Carnaille B, et al. 2017a European Society of Endocrine Surgeons (ESES) and European Network for the Study of Adrenal Tumours (ENSAT) recommendations for the surgical management of adrenocortical carcinoma. Br J Surg 104 358–376. (10.1002/BJS.10414) 28199015

[bib77] Gaujoux S, Weinandt M, Bonnet S, et al. 2017b Surgical treatment of adrenal carcinoma. J Visc Surg 154 335–343. (10.1016/J.JVISCSURG.2017.06.010) 28754418

[bib78] Ginsburg KB, Chandra AA, Handorf EA, et al. 2022 Association of surgical approach with treatment burden, oncological effectiveness, and perioperative morbidity in adrenocortical carcinoma. Clin Genitourin Cancer 20 497.e1–497.e7. (10.1016/J.CLGC.2022.04.011) PMC10027416 35618598

[bib79] Giordano TJ, Kuick R, Else T, et al. 2009 Molecular classification and prognostication of adrenocortical tumors by transcriptome profiling. Clin Cancer Res 15 668–676. (10.1158/1078-0432.CCR-08-1067) 19147773 PMC2629378

[bib80] Grande E, Benavent Viñuales M, Molina-Cerrillo J, et al. 2024 Cabozantinib plus atezolizumab in locally advanced/metastatic adrenocortical carcinoma: results from a multi-cohort basket phase II trial, CABATEN/GETNE-T1914. J Clin Oncol 42 (Supplement 4) 1. (10.1200/JCO.2024.42.4_SUPPL.1)37847871

[bib81] Habra MA, Ejaz S, Feng L, et al. 2013 A retrospective cohort analysis of the efficacy of adjuvant radiotherapy after primary surgical resection in patients with adrenocortical carcinoma. J Clin Endocrinol Metab 98 192–197. (10.1210/JC.2012-2367) 23150683 PMC3537094

[bib82] Habra MA, Stephen B, Campbell M, et al. 2019 Phase II clinical trial of pembrolizumab efficacy and safety in advanced adrenocortical carcinoma. J ImmunoTherapy Cancer 7 253. (10.1186/s40425-019-0722-x) PMC6751592 31533818

[bib83] Hahner S, Kreissl MC, Fassnacht M, et al. 2012 [131I]iodometomidate for targeted radionuclide therapy of advanced adrenocortical carcinoma. J Clin Endocrinol Metab 97 914–922. (10.1210/JC.2011-2765) 22170726

[bib84] Hahner S, Hartrampf PE, Mihatsch PW, et al. 2022 Targeting 11-beta hydroxylase with [131I]IMAZA: a novel approach for the treatment of advanced adrenocortical carcinoma. J Clin Endocrinol Metab 107 E1348–E1355. (10.1210/CLINEM/DGAB895) 34904171

[bib85] Haluska P, Worden F, Olmos D, et al. 2010 Safety, tolerability, and pharmacokinetics of the anti-IGF-1R monoclonal antibody figitumumab in patients with refractory adrenocortical carcinoma. Cancer Chemother Pharmacol 65 765–773. (10.1007/S00280-009-1083-9) 19649631 PMC2875253

[bib86] Hamblin R, Coulden A, Fountas A, et al. 2022 The diagnosis and management of Cushing’s syndrome in pregnancy. J Neuroendocrinol 34 e13118. (10.1111/jne.13118) 35491087 PMC9541401

[bib186] Head L, Kiseljak-Vassiliades K, Clark TJ, et al. 2019 Response to immunotherapy in combination with mitotane in patients with metastatic adrenocortical cancer. J Endocr Soc 3 2295–2304. (10.1210/js.2019-00305) 31745526 PMC6853671

[bib87] Henning JEK, Deutschbein T, Altieri B, et al. 2017 Gemcitabine-based chemotherapy in adrenocortical carcinoma: a multicenter study of efficacy and predictive factors. J Clin Endocrinol Metab 102 4323–4332. (10.1210/jc.2017-01624) 29092062

[bib88] Herrmann LJM, Heinze B, Fassnacht M, et al. 2012 TP53 germline mutations in adult patients with adrenocortical carcinoma. J Clin Endocrinol Metab 97 E476–E485. (10.1210/JC.2011-1982) 22170717

[bib89] Ho J, Turkbey B, Edgerly M, et al. 2013 Role of radiotherapy in adrenocortical carcinoma. Cancer J 19 288–294. (10.1097/PPO.0B013E31829E3221) 23867507 PMC8381259

[bib90] Hu X, Yang WX, Shao YX, et al. 2020 Minimally invasive versus open adrenalectomy in patients with adrenocortical carcinoma: a meta-analysis. Ann Surg Oncol 27 3858–3869. (10.1245/S10434-020-08454-1) 32277316

[bib91] Ilias I, Sahdev A, Reznek RH, et al. 2007 The optimal imaging of adrenal tumours: a comparison of different methods. Endocr Relat Cancer 14 587–599. (10.1677/ERC-07-0045) 17914090

[bib92] Kastelan D, Muzurovic E & Dusek T 2021 Approach to patients with European Network for the study of adrenal tumor stages I and II adrenocortical carcinomas. Curr Opin Endocrinol Diabetes Obes 28 265–270. (10.1097/MED.0000000000000626) 33709971

[bib93] Kenney L & Hughes M 2023 Adrenocortical carcinoma: role of adjuvant and neoadjuvant therapy. Surg Oncol Clin N Am 32 279–287. (10.1016/J.SOC.2022.10.005) 36925185

[bib94] Kerkhofs TMA, Verhoeven RHA, Van Der Zwan JM, et al. 2013 Adrenocortical carcinoma: a population-based study on incidence and survival in The Netherlands since 1993. Eur J Cancer 49 2579–2586. (10.1016/j.ejca.2013.02.034) 23561851

[bib95] Khan TS, Sundin A, Juhlin C, et al. 2004 Vincristine, cisplatin, teniposide, and cyclophosphamide combination in the treatment of recurrent or metastatic adrenocortical cancer. Med Oncol 21 167–178. (10.1385/MO:21:2:167) 15299189

[bib96] Khosla D, Kapoor R, Singla AK, et al. 2023 Treatment outcomes of adjuvant radiotherapy in adrenocortical carcinoma – a 13-years experience from a tertiary care centre. Rare Tumors 15 20363613231160699. (10.1177/20363613231160699) 36860827 PMC9969472

[bib97] Kimpel O, Bedrose S, Megerle F, et al. 2021 Adjuvant platinum-based chemotherapy in radically resected adrenocortical carcinoma: a cohort study. Br J Cancer 125 1233–1238. (10.1038/S41416-021-01513-8) 34400803 PMC8548516

[bib98] Kimpel O, Altieri B, Dischinger U, et al. 2023 Early detection of recurrence and progress using serum steroid profiling by LC-MS/MS in patients with adrenocortical carcinoma. Metabolites 14 20. (10.3390/METABO14010020) 38248823 PMC10819433

[bib99] Kimpel O, Altieri B, Laganà M, et al. 2024 The value of local therapies in advanced adrenocortical carcinoma. Cancers 16 706. (10.3390/CANCERS16040706) 38398097 PMC10886520

[bib100] Klein O, Senko C, Carlino MS, et al. 2021 Combination immunotherapy with ipilimumab and nivolumab in patients with advanced adrenocortical carcinoma: a subgroup analysis of CA209-538. OncoImmunology 10 1908771. (10.1080/2162402X.2021.1908771) 33889439 PMC8043165

[bib101] Konopleva M, Martinelli G, Daver N, et al. 2020 MDM2 inhibition: an important step forward in cancer therapy. Leukemia 34 2858–2874. (10.1038/S41375-020-0949-Z) 32651541

[bib102] Koo BK, Van Es JH, Van Den Born M, et al. 2015 Porcupine inhibitor suppresses paracrine Wnt-driven growth of Rnf43;Znrf3-mutant neoplasia. Proc Natl Acad Sci U S A 112 7548–7550. (10.1073/PNAS.1508113112) 26023187 PMC4475934

[bib103] Kroiss M, Quinkler M, Lutz WK, et al. 2011 Drug interactions with mitotane by induction of CYP3A4 metabolism in the clinical management of adrenocortical carcinoma. Clin Endocrinol 75 585–591. (10.1111/J.1365-2265.2011.04214.X) 21883349

[bib104] Kroiss M, Quinkler M, Johanssen S, et al. 2012 Sunitinib in refractory adrenocortical carcinoma: a phase II, single-arm, open-label trial. J Clin Endocrinol Metab 97 3495–3503. (10.1210/JC.2012-1419) 22837187

[bib105] Kwon D, Rah CS, Kim BC, et al. 2024 Early stage adrenocortical carcinoma-what contributes to poor prognosis after adrenalectomy? A retrospective cohort study. Ann Surg Treat Res 107 187–194. (10.4174/ASTR.2024.107.4.187) 39416880 PMC11473319

[bib106] Laganà M, Grisanti S, Ambrosini R, et al. 2022 Phase II study of cabazitaxel as second-third line treatment in patients with metastatic adrenocortical carcinoma. ESMO Open 7 100422. (10.1016/j.esmoop.2022.100422) 35272132 PMC9058897

[bib107] Le Tourneau C, Hoimes C, Zarwan C, et al. 2018 Avelumab in patients with previously treated metastatic adrenocortical carcinoma: phase 1b results from the JAVELIN solid tumor trial. J Immunother Cancer 6 111. (10.1186/s40425-018-0424-9) 30348224 PMC6198369

[bib108] Leal LF, Bueno AC, Gomes DC, et al. 2015 Inhibition of the Tcf/beta-catenin complex increases apoptosis and impairs adrenocortical tumor cell proliferation and adrenal steroidogenesis. Oncotarget 6 43016–43032. (10.18632/ONCOTARGET.5513) 26515592 PMC4767488

[bib109] Leboulleux S, Deandreis D, Al Ghuzlan A, et al. 2010 Adrenocortical carcinoma: is the surgical approach a risk factor of peritoneal carcinomatosis? Eur J Endocrinol 162 1147–1153. (10.1530/EJE-09-1096) 20348273

[bib110] Lee JH, Faderl S, Pagel JM, et al. 2020 Phase 1 study of CWP232291 in patients with relapsed or refractory acute myeloid leukemia and myelodysplastic syndrome. Blood Adv 4 2032–2043. (10.1182/BLOODADVANCES.2019000757) 32396615 PMC7218422

[bib111] Lerario AM, Worden FP, Ramm CA, et al. 2014 The combination of insulin-like growth factor receptor 1 (IGF1R) antibody cixutumumab and mitotane as a first-line therapy for patients with recurrent/metastatic adrenocortical carcinoma: a multi-institutional NCI-sponsored trial. Horm Cancer 5 232–239. (10.1007/S12672-014-0182-1) 24849545 PMC4298824

[bib112] Liang R, Weigand I, Lippert J, et al. 2020 Targeted gene expression profile reveals CDK4 as therapeutic target for selected patients with adrenocortical carcinoma. Front Endocrinol 11 219. (10.3389/FENDO.2020.00219) PMC7176906 32373071

[bib113] Libé R, Borget I, Ronchi CL, et al. 2015 Prognostic factors in stage III-IV adrenocortical carcinomas (ACC): an European Network for the Study of Adrenal Tumor (ENSAT) study. Ann Oncol 26 2119–2125. (10.1093/ANNONC/MDV329) 26392430

[bib114] Libé R, Pais A, Violon F, et al. 2023 Positive correlation between 18 F-FDG uptake and tumor-proliferating antigen Ki-67 expression in adrenocortical carcinomas. Clin Nucl Med 48 381–386. (10.1097/RLU.0000000000004593) 36758555

[bib115] Livhits M, Li N, Yeh MW, et al. 2014 Surgery is associated with improved survival for adrenocortical cancer, even in metastatic disease. Surgery 156 1531–1541. (10.1016/J.SURG.2014.08.047) 25456949 PMC5031479

[bib116] Lombardi CP, Raffaelli M, De Crea C, et al. 2012 Open versus endoscopic adrenalectomy in the treatment of localized (stage I/II) adrenocortical carcinoma: results of a multiinstitutional Italian survey. Surgery 152 1158–1164. (10.1016/J.SURG.2012.08.014) 23068084

[bib117] Luconi M, Mangoni M, Gelmini S, et al. 2010 Rosiglitazone impairs proliferation of human adrenocortical cancer: preclinical study in a xenograft mouse model. Endocr Relat Cancer 17 169–177. (10.1677/ERC-09-0170) 19955217

[bib187] Ma G, Zhang X, Wang M, et al. 2021 Role of 18F-FDG PET/CT in the differential diagnosis of primary benign and malignant unilateral adrenal tumors. Quant Imaging Med Surg 11 2013–2018. (10.21037/qims-20-875) 33936982 PMC8047365

[bib118] Marty M, Gaye D, Perez P, et al. 2018 Diagnostic accuracy of computed tomography to identify adenomas among adrenal incidentalomas in an endocrinological population. Eur J Endocrinol 178 439–446. (10.1530/EJE-17-1056) 29467231

[bib119] Maurice MJ, Bream MJ, Kim SP, et al. 2017 Surgical quality of minimally invasive adrenalectomy for adrenocortical carcinoma: a contemporary analysis using the National Cancer Database. BJU Int 119 436–443. (10.1111/BJU.13618) 27488744

[bib120] McCoy K, Valdez C & Gibson CE 2022 Retroperitoneoscopic adrenalectomy: indications and technical considerations. Laparosc Surg 6 6–13. (10.21037/LS-21-24/COIF)

[bib121] McGregor BA, Campbell MT, Xie W, et al. 2021 Results of a multicenter , phase 2 study of nivolumab and ipilimumab for patients with advanced rare genitourinary malignancies. Cancer 127 840–849. (10.1002/cncr.33328) 33216356 PMC13213840

[bib122] Megerle F, Herrmann W, Schloetelburg W, et al. 2018 Mitotane monotherapy in patients with advanced adrenocortical carcinoma. J Clin Endocrinol Metab 103 1686–1695. (10.1210/JC.2017-02591) 29452402

[bib123] Mehlich A, Bolanowski M, Mehlich D, et al. 2023 Medical treatment of Cushing’s disease with concurrent diabetes mellitus. Front Endocrinol 14 1174119. (10.3389/FENDO.2023.1174119) PMC10150952 37139336

[bib124] Memeh K, Abou Azar S, Afolaranmi O, et al. 2025 Survival impact of treatment utilization and margin status after resection of adrenocortical carcinoma. Am J Surg 239 115999. (10.1016/J.AMJSURG.2024.115999) 39427460

[bib125] Mete O, Asa SL, Giordano TJ, et al. 2018 Immunohistochemical biomarkers of adrenal cortical neoplasms. Endocr Pathol 29 137–149. (10.1007/S12022-018-9525-8) 29542002

[bib126] Mete O, Erickson LA, Juhlin CC, et al. 2022 Overview of the 2022 WHO classification of adrenal cortical tumors. Endocr Pathol 33 155–196. (10.1007/S12022-022-09710-8) 35288842 PMC8920443

[bib127] Michalski K, Schlötelburg W, Hartrampf PE, et al. 2023 Radiopharmaceuticals for treatment of adrenocortical carcinoma. Pharmaceuticals 17 25. (10.3390/PH17010025) 38256859 PMC10820941

[bib128] Mínguez Ojeda C, Gómez Dos Santos V, Álvaro Lorca J, et al. 2022 Tumour size in adrenal tumours: its importance in the indication of adrenalectomy and in surgical outcomes-a single-centre experience. J Endocrinol Investig 45 1999–2006. (10.1007/S40618-022-01836-0) 35748977

[bib129] Morris JA, Campbell P, Xu L, et al. 2023 Cushing syndrome due to adrenocortical carcinoma during pregnancy. JCEM Case Rep 1 luad118. (10.1210/JCEMCR/LUAD118) 38021076 PMC10652247

[bib130] Naing A, Lorusso P, Fu S, et al. 2013 Insulin growth factor receptor (IGF-1R) antibody cixutumumab combined with the mTOR inhibitor temsirolimus in patients with metastatic adrenocortical carcinoma. Br J Cancer 108 826–830. (10.1038/BJC.2013.46) 23412108 PMC3590681

[bib131] Neumann HPH, Tsoy U, Bancos I, et al. 2019 Comparison of pheochromocytoma-specific morbidity and mortality among adults with bilateral pheochromocytomas undergoing total adrenalectomy vs cortical-sparing adrenalectomy. JAMA Netw Open 2 e198898. (10.1001/jamanetworkopen.2019.8898) 31397861 PMC6692838

[bib132] Nieman LK, Biller BMK, Findling JW, et al. 2015 Treatment of cushing’s syndrome: an endocrine society clinical practice guideline. J Clin Endocrinol Metab 100 2807–2831. (10.1210/jc.2015-1818) 26222757 PMC4525003

[bib133] O’Sullivan C, Edgerly M, Velarde M, et al. 2014 The VEGF inhibitor axitinib has limited effectiveness as a therapy for adrenocortical cancer. J Clin Endocrinol Metab 99 1291–1297. (10.1210/JC.2013-2298) 24423320 PMC3973787

[bib134] Owen DH, Patel S, Wei L, et al. 2019 Metastatic adrenocortical carcinoma: a single institutional experience. Horm Cancer 10 161–167. (10.1007/S12672-019-00367-0) 31468469 PMC10355711

[bib136] Papadopoulos KP, El-Rayes BF, Tolcher AW, et al. 2017 A Phase 1 study of ARQ 087, an oral pan-FGFR inhibitor in patients with advanced solid tumours. Br J Cancer 117 1592–1599. (10.1038/BJC.2017.330) 28972963 PMC5729432

[bib137] Petersenn S, Richter PA, Broemel T, et al. 2015 Computed tomography criteria for discrimination of adrenal adenomas and adrenocortical carcinomas: analysis of the German ACC registry. Eur J Endocrinol 172 415–422. (10.1530/EJE-14-0916) 25599706

[bib138] Petr EJ & Else T 2016 Genetic predisposition to endocrine tumors: diagnosis, surveillance and challenges in care. Semin Oncol 43 582–590. (10.1053/j.seminoncol.2016.08.007) 27899191

[bib139] Pittaway JFH, Lipsos C, Mariniello K, et al. 2021 The role of delta-like non-canonical Notch ligand 1 (DLK1) in cancer. Endocr Relat Cancer 28 R271–R287. (10.1530/ERC-21-0208) 34627131

[bib140] Puglisi S, Perotti P, Cosentini D, et al. 2018a Decision-making for adrenocortical carcinoma: surgical, systemic, and endocrine management options. Expet Rev Anticancer Ther 18 1125–1133. (10.1080/14737140.2018.1510325) 30117750

[bib141] Puglisi S, Perotti P, Pia A, et al. 2018b Adrenocortical carcinoma with hypercortisolism. Endocrinol Metab Clin N Am 47 395–407. (10.1016/J.ECL.2018.02.003) 29754640

[bib142] Puglisi S, Basile V, Sperone P, et al. 2023a Pregnancy in patients with adrenocortical carcinoma: a case-based discussion. Rev Endocr Metab Disord 24 85–96. (10.1007/S11154-022-09769-Y) 36414840

[bib143] Puglisi S, Calabrese A, Ferraù F, et al. 2023b New findings on presentation and outcome of patients with adrenocortical cancer: results from a National Cohort Study. J Clin Endocrinol Metab 108 2517–2525. (10.1210/CLINEM/DGAD199) 37022947

[bib144] Quinkler M, Hahner S, Wortmann S, et al. 2008 Treatment of advanced adrenocortical carcinoma with erlotinib plus gemcitabine. J Clin Endocrinol Metab 93 2057–2062. (10.1210/JC.2007-2564) 18334586

[bib145] Raj N, Zheng Y, Kelly V, et al. 2020 PD-1 blockade in advanced adrenocortical carcinoma. J Clin Oncol 38 71–80. (10.1200/JCO.19.01586) 31644329 PMC7351334

[bib146] Raymond VM, Everett JN, Furtado LV, et al. 2013 Adrenocortical carcinoma is a lynch syndrome-associated cancer. J Clin Oncol 31 3012–3018. (10.1200/JCO.2012.48.0988) 23752102 PMC3739861

[bib147] Remde H, Schmidt-Pennington L, Reuter M, et al. 2023 Outcome of immunotherapy in adrenocortical carcinoma: a retrospective cohort study. Eur J Endocrinol 188 485–493. (10.1093/EJENDO/LVAD054) 37260092

[bib148] Rodriguez-Galindo C, Krailo MD, Pinto EM, et al. 2021 Treatment of pediatric adrenocortical carcinoma with surgery, retroperitoneal lymph node dissection, and chemotherapy: the Children’s Oncology Group ARAR0332 Protocol. J Clin Oncol 39 2463–2473. (10.1200/JCO.20.02871) 33822640 PMC8462560

[bib149] Romanisio M, Daffara T, Pitino R, et al. 2024 [18F]FDG-PET/CT in adrenal lesions: diagnostic performance in different clinical settings. Endocrine 87 325–333. (10.1007/S12020-024-04042-5) 39294519 PMC11739187

[bib150] Roux C, Boileve A, Faron M, et al. 2022 Loco-regional therapies in oligometastatic adrenocortical carcinoma. Cancers 14 2730. (10.3390/CANCERS14112730) 35681708 PMC9179919

[bib151] Sabolch A, Else T, Griffith KA, et al. 2015 Adjuvant radiation therapy improves local control after surgical resection in patients with localized adrenocortical carcinoma. Int J Radiat Oncol Biol Phys 92 252–259. (10.1016/J.IJROBP.2015.01.007) 25754631

[bib152] Samnotra V, Vassilopoulou-Sellin R, Fojo AT, et al. 2007 A phase II trial of gefitinib monotherapy in patients with unresectable adrenocortical carcinoma (ACC). J Clin Oncol 25 (Supplement 18) 15527. (10.1200/JCO.2007.25.18_SUPPL.15527)

[bib153] Sarvestani AL, Gregory SN, Teke ME, et al. 2023 Mitotane with or without cisplatin and etoposide for patients with a high risk of recurrence in stages 1-3 adrenocortical cancer after surgery. Ann Surg Oncol 30 680–682. (10.1245/S10434-022-12725-4) 36305989

[bib154] Schloetelburg W, Ebert I, Petritsch B, et al. 2022 Adrenal wash-out CT: moderate diagnostic value in distinguishing benign from malignant adrenal masses. Eur J Endocrinol 186 183–193. (10.1530/EJE-21-0650) PMC8679842 34813495

[bib155] Schloetelburg W, Hartrampf PE, Kosmala A, et al. 2024 Predictive value of C-X-C motif chemokine receptor 4-directed molecular imaging in patients with advanced adrenocortical carcinoma. Eur J Nucl Med Mol Imag 51 3643–3650. (10.1007/S00259-024-06800-Z) PMC11445370 38896128

[bib156] Shin YR & Kim KA 2015 Imaging features of various adrenal neoplastic lesions on radiologic and nuclear medicine imaging. AJR Am J Roentgenol 205 554–563. (10.2214/AJR.15.14467) 26295641

[bib157] Sinclair TJ, Gillis A, Alobuia WM, et al. 2020 Surgery for adrenocortical carcinoma: when and how? Best Pract Res Clin Endocrinol Metabol 34 101408. (10.1016/j.beem.2020.101408) 32265101

[bib158] Solomon VR, Alizadeh E, Bernhard W, et al. 2019 111In- and 225Ac-labeled cixutumumab for imaging and α-particle radiotherapy of IGF-1R positive triple-negative breast cancer. Mol Pharm 16 4807–4816. (10.1021/ACS.MOLPHARMACEUT.9B00542) 31518138

[bib159] Solomon VR, Alizadeh E, Bernhard W, et al. 2020 Development and preclinical evaluation of cixutumumab drug conjugates in a model of insulin growth factor receptor I (IGF-1R) positive cancer. Sci Rep 10 18549. (10.1038/S41598-020-75279-Z) 33122707 PMC7596529

[bib160] Sperone P, Ferrero A, Daffara F, et al. 2010 Gemcitabine plus metronomic 5-fluorouracil or capecitabine as a second-/third-line chemotherapy in advanced adrenocortical carcinoma: a multicenter phase II study. Endocr Relat Cancer 17 445–453. (10.1677/ERC-09-0281) 20410174

[bib161] Stoinis N, Creeper K, Phillips J, et al. 2024 Diverse presentations of Cushing’s syndrome during pregnancy – a case series. Aust N Z J Obstet Gynaecol 64 314–318. (10.1111/AJO.13793) 38284434

[bib135] Szkodziak P, Szkodziak F, Korolczuk A, et al. 2024 The effect of adjuvant mitotane therapy of the adrenocortical carcinoma on the endometrium and its clinical consequences in menstruating women. Literature review and authors’ own experiences. Am J Cancer Res 14 1802–1814. (10.62347/QKWF9884) 38726272 PMC11076245

[bib162] Tabarin A, Haissaguerre M, Lassole H, et al. 2022 Efficacy and tolerance of osilodrostat in patients with Cushing’s syndrome due to adrenocortical carcinomas. Eur J Endocrinol 186 K1–K4. (10.1530/EJE-21-1008) 34905500

[bib163] Terzolo M, Fassnacht M, Perotti P, et al. 2023 Adjuvant mitotane versus surveillance in low-grade, localised adrenocortical carcinoma (ADIUVO): an international, multicentre, open-label, randomised, phase 3 trial and observational study. Lancet Diabetes Endocrinol 11 720–730. (10.1016/S2213-8587(23)00193-6) 37619579 PMC10522778

[bib164] Tripto-Shkolnik L, Blumenfeld Z, Bronshtein M, et al. 2013 Pregnancy in a patient with adrenal carcinoma treated with mitotane: a case report and review of literature. J Clin Endocrinol Metab 98 443–447. (10.1210/JC.2012-2839) 23275528

[bib165] Turla A, Laganà M, Grisanti S, et al. 2022 Supportive therapies in patients with advanced adrenocortical carcinoma submitted to standard EDP-M regimen. Endocrine 77 438–443. (10.1007/S12020-022-03075-Y) 35567656 PMC9385801

[bib166] Urup T, Pawlak WZ, Petersen PM, et al. 2013 Treatment with docetaxel and cisplatin in advanced adrenocortical carcinoma, a phase II study. Br J Cancer 108 1994–1997. (10.1038/BJC.2013.229) 23652308 PMC3670472

[bib167] Van Slooten H & Van Oosterom AT 1983 CAP (cyclophosphamide, doxorubicin, and cisplatin) regimen in adrenal cortical carcinoma. Cancer Treat Rep 67 377–379. (https://pubmed.ncbi.nlm.nih.gov/6687835/) 6687835

[bib168] Varlamov EV, Han AJ & Fleseriu M 2021 Updates in adrenal steroidogenesis inhibitors for Cushing’s syndrome – a practical guide. Best Pract Res Clin Endocrinol Metabol 35 101490. (10.1016/J.BEEM.2021.101490) 33707082

[bib169] Veltri A, Basile D, Calandri M, et al. 2020 Oligometastatic adrenocortical carcinoma: the role of image-guided thermal ablation. Eur Radiol 30 6958–6964. (10.1007/S00330-020-07019-W) 32621242

[bib170] Vogg N, Müller T, Floren A, et al. 2023 Simplified urinary steroid profiling by LC-MS as diagnostic tool for malignancy in adrenocortical tumors. Clin Chim Acta 543 117301. (10.1016/J.CCA.2023.117301) 36948238

[bib171] Wagle N, Grabiner BC, Van Allen EM, et al. 2014 Activating mTOR mutations in a patient with an extraordinary response on a phase I trial of everolimus and pazopanib. Cancer Discov 4 546–553. (10.1158/2159-8290.CD-13-0353) 24625776 PMC4122326

[bib172] Wang Z, Hu H, Heitink L, et al. 2023 The anti-cancer agent APR-246 can activate several programmed cell death processes to kill malignant cells. Cell Death Differ 30 1033–1046. (10.1038/S41418-023-01122-3) 36739334 PMC10070280

[bib173] Weigel B, Malempati S, Reid JM, et al. 2014 Phase 2 trial of cixutumumab in children, adolescents, and young adults with refractory solid tumors: a report from the Children’s Oncology Group. Pediatr Blood Cancer 61 452–456. (10.1002/PBC.24605) 23956055 PMC4511811

[bib174] Weiss LM, Medeiros LJ & Vickery AL 1989 Pathologic features of prognostic significance in adrenocortical carcinoma. Am J Surg Pathol 13 202–206. (10.1097/00000478-198903000-00004) 2919718

[bib175] Wieneke JA, Thompson LDR & Heffess CS 2003 Adrenal cortical neoplasms in the pediatric population: a clinicopathologic and immunophenotypic analysis of 83 patients. Am J Surg Pathol 27 867–881. (10.1097/00000478-200307000-00001) 12826878

[bib176] Williamson SK, Lew D, Miller GJ, et al. 2000 Phase II evaluation of cisplatin and etoposide followed by mitotane at disease progression in patients with locally advanced or metastatic adrenocortical carcinoma: a Southwest Oncology Group study. Cancer 88 1159–1165. (10.1002/(SICI)1097-0142(20000301)88:5<1159::AID-CNCR28>3.0.CO;2-R) 10699907

[bib177] Wong KK, Miller BS, Viglianti BL, et al. 2016 Molecular imaging in the management of adrenocortical cancer: a systematic review. Clin Nucl Med 41 e368–e382. (10.1097/RLU.0000000000001112) 26825212

[bib178] Wood BJ, Abraham J, Hvizda JL, et al. 2003 Radiofrequency ablation of adrenal tumors and adrenocortical carcinoma metastases. Cancer 97 554–560. (10.1002/CNCR.11084) 12548596 PMC2443414

[bib179] Wortmann S, Quinkler M, Ritter C, et al. 2010 Bevacizumab plus capecitabine as a salvage therapy in advanced adrenocortical carcinoma. Eur J Endocrinol 162 349–356. (10.1530/EJE-09-0804) 19903796

[bib180] Wu K, Liu Z, Liang J, et al. 2018 Laparoscopic versus open adrenalectomy for localized (stage 1/2) adrenocortical carcinoma: experience at a single, high-volumecenter. Surgery 164 1325–1329. (10.1016/J.SURG.2018.07.026) 30266443

[bib181] Zhang CD & Ioachimescu AG 2024 Challenges in the postsurgical recovery of cushing syndrome: glucocorticoid withdrawal syndrome. Front Endocrinol 15 1353543. (10.3389/FENDO.2024.1353543) PMC11045978 38681763

[bib182] Zheng S, Cherniack AD, Dewal N, et al. 2016 Comprehensive pan-genomic characterization of adrenocortical carcinoma. Cancer Cell 29 723–736. (10.1016/j.ccell.2016.04.002) 27165744 PMC4864952

[bib183] Zhu Y, Wang M, Zhao X, et al. 2017 Rottlerin as a novel chemotherapy agent for adrenocortical carcinoma. Oncotarget 8 22825–22834. (10.18632/ONCOTARGET.15221) 28423559 PMC5410265

[bib184] Zhu J, Zheng Z, Shen J, et al. 2020 Efficacy of adjuvant radiotherapy for treatment of adrenocortical carcinoma: a retrospective study and an updated meta-analysis. Radiat Oncol 15 118. (10.1186/S13014-020-01533-3) 32448148 PMC7245885

[bib185] Zhu Y-C, Wei Z-G, Wang J-J, et al. 2024 Camrelizumab plus apatinib for previously treated advanced adrenocortical carcinoma: a single-arm phase 2 trial. Nat Commun 15 10371. (10.1038/S41467-024-54661-9) 39609453 PMC11604670

