# Supplementary material for: Spanish consensus on the diagnosis and management of adrenocortical carcinoma
Source: Endocr Relat Cancer. 2025 Apr 24;32(5):e250034. doi: 10.1530/ERC-25-0034 (PMC12053981; doi:10.1530/ERC-25-0034)
Supplement: Supplementary file 1 [file supplementary_materials.pdf]

Supplementary material

S1. Open and laparoscopic approach in localized ACC

The increasing diagnosis of incidental adrenal masses treated with a minimally invasive surgery (MIS) implies an increased risk of an unexpected histologic diagnosis of ACC in up to 10% of patients. In the absence of radiological evidence of loco-regional invasion, lymph node involvement, venous thrombosis or distant metastasis, it is difficult to predict the presence of a malignant adrenal lesion (Bellantone et al., 2015; Datta & Roses, 2016; Sinclair et al., 2020). MIS adrenalectomy is the preferred technique in the treatment of benign adrenal masses smaller than 6 cm; however, the role of adrenalectomy in ACC remains controversial (Autorino et al., 2016; Dickson et al., 2018; Maurice et al., 2017). The oncologic results of laparoscopic adrenalectomy are inconclusive. Classically, laparoscopic adrenalectomy has been associated with a higher rate of positive margins, peritoneal carcinomatosis and local recurrences; however, in experienced centers and in current series, the oncologic results of the laparoscopic approach are not inferior to those of open surgery (Cooper et al., 2013; Donatini et al., 2014; Leboulleux et al., 2010; Lombardi, Raffaelli, De Crea, et al., 2012; Mirallié et al., 2019). Several studies have shown the feasibility of laparoscopic surgery for ACC with equivalent results in terms of the quality of the excision (margins, lymph node dissection) and overall survival without recurrence compared to laparotomy. Nevertheless, and despite these results, the debate on the approach remains, and no consensus has been reached. In a recent 2018 study, Wu et al (Wu et al., 2018) studied the results and follow-up of patients who underwent operations for stage I and II ACC. This was a retrospective monocentric study from 2009 to 2017. Survival and survival without recurrence were studied. The two groups (laparotomy n = 23/laparoscopy n = 21) were comparable. The oncological results for a period of observation and monitoring that was identical for the two groups showed an identical overall rate ($p = 0.989$) of local, peritoneal and metastatic recurrences (52% in the two groups). The time of the recurrence's appearance was not significantly different ($p = 0.564$) at 22 months in the laparotomy group and 25 months in the laparoscopy group. On the other hand, there was a significant difference ($p = 0.035$) in terms of local and peritoneal recurrences with 42% for the laparoscopy group versus 22% for the laparotomy group. Lastly, the overall survival (43% versus 47%; $p = 0.635$) and median survival without recurrence (36% versus 39%; $p = 0.802$) was not significantly different between the two groups. The authors concluded that although overall development in terms of survival and metastatic development are more due to the tumor's biology, local and peritoneal recurrences are more directly impacted by the surgical techniques themselves and that therefore, the results of the study suggest that excision by laparotomy must remain the norm for a proven or highly suspected ACC.

Controversy reaches the recommendations of clinical guidelines. The American Society of Gastrointestinal and Endoscopic Surgeons recommend open surgery in those patients with suspected adrenal carcinoma as do the NCCN guidelines. The European Society of Endocrine Surgeons state that laparoscopic adrenalectomy can be performed in potentially malignant lesions is stage I-II and size < 10 cm. Similarly, the European Society of Medical Oncology considers laparoscopic adrenalectomy safe and effective for a selected group of patients with lesions < 8 cm without preoperative evidence of loco-regional invasion (S. Gaujoux et al., 2017)

According to the anatomic reviews, lymphatic mapping of adrenal gland is difficult and complex. There are 2 principals drainage flows: posterior to the inferior cava vein and right/left edge of the aorta and anterior downward to the lumbo-aortic nodes and the inter-aorto-caval space. Description of the pathways appears difficult in clinical practice and impossible to predict for a malignant adrenal tumor (Sébastien Gaujoux & Brennan, 2012). Regional lymphadenectomy in the course of primary surgery not only allows more accurate staging but has been shown to significantly reduce local recurrences and cancer-specific mortality (Gerry et al., 2016; Reibetanz et al., 2019). However, although there is no definitive agreement on the extent of lymphadenectomy, this should include, at least, periadrenal/perirenal fat and renal hilum. Routinely, dissection of paraaortic and/or paracaval lymph nodes ipsilateral to the tumor is still debated without preoperartive imaging showing node involvement (Wu et al., 2018). Lymph node involvement significantly reduces overall survival: 19% of N + patients were alive after five years compared to 63% of N − patients. In a multivariate analysis, lymph node involvement remained a predictive factor of mortality (relative risk: 2.06, P = 0.0048). Another important piece of information in this study is the definition of the minimum number of nodes that have to be resected to assure the lymph node status. This minimum number was established in 432.

A specific consideration in adrenal surgery for carcinoma is the possibility of tumor vascular involvement, which occurs in up to 25% of cases. Although vascular invasion carries a poor prognosis, it is not a contraindication for surgery, although it requires careful preoperative planning that considers the location and extent of the tumor thrombus (Annamaria et al., 2015).

Both laparoscopic and open approaches to adrenalectomy are considered safe. Several large national studies published recently documented an overall complication rate of 11-18% for adrenalectomies, including both laparoscopic and open cases. A study directly comparing postoperative outcomes after resection of benign vs. malignant adrenal tumors demonstrated higher rates of complications associated with malignant disease in terms of intraoperative vascular and splenic injuries, as well as higher rates of postoperative complications including hematoma, adrenocortical insufficiency, venous thromboembolism, pneumothorax, shock, and

cardiac complications compared to patients who underwent resection for benign disease (Hauch et al., 2015; Marcadis et al., 2017). It has been repeatedly demonstrated that adrenalectomies performed by high-volume surgeons are associated with a lower complication rate (Langenhuijsen et al., 2016; Lombardi, Raffaelli, Boniardi, et al., 2012). A thorough pre-operative endocrine work-up should be undertaken in all patients with suspected ACC11. This is not only to rule out pheochromocytoma, but also to assess the perioperative risks associated with cortisol-secreting ACCs. Appropriate precautions must be taken to manage post-operative adrenal insufficiency.

Pathological analysis of the adrenalectomy specimen allows analysis of the most important prognostic factors: tumor stage, resection status (R0-R1-R2) and Ki-67% index. Hypercortisolism and the patient's functional status are added to these pathological factors. The pathological prognostic factors allow patients to be classified into 2 risk groups: low/moderate risk ACC includes stage I–II and R0 and Ki67 ≤10%, whereas high-risk ACC includes stage III, R1 or Ki67 >10%. Mitotane could be recommended in those patients without macroscopic residual tumor after surgery but have a high risk of recurrence. However, adjuvant therapy is controversial for patients at low/moderate risk of recurrence (stage I–II, R0 resection and Ki67 ≤10%), in these cases adjuvant therapy options should be discussed on an individual basis. Use of radiation therapy in addition to mitotane should also be individualized in patients with R1 or Rx resection or in stage III (Fassnacht et al., 2018; Kenney & Hughes, 2023; Terzolo et al., 2023).

S2. Treatment of ACC with other steroidogenesis inhibitors different than mitotane

1. Metyrapone

Metyrapone interferes with the final step of cortisol biosynthesis by inhibiting 11β-hydroxylase (CYP11B1) mainly, leading to an accumulation of 11-deoxycortisol and a reduction in cortisol levels. It is commonly used when mitotane is not indicated, not well-tolerated or insufficient to control Cushing ´syndrome (Turla et al., 2022). Metyrapone's pharmacokinetics are not influenced by concurrent mitotane use, and the combination of EDP-M with metyrapone can result in effective and rapid control of Cushing's syndrome (Claps et al., 2018). Due to its short half-life (~2 hours), multiple daily doses are required, starting at 250 mg every 6 hours, adjusting to a daily dose ranging from 500 to 3000 mg. 11β-hydroxylase inhibitors may lead to increased mineralocorticoid precursors that need surveillance for hypertension and increase in androgen levels due to accumulation of precursor metabolites that shift to the androgen synthesis pathway, increasing hirsutism in women may be an undesirable side effect.

2. Osilodrostat

Osilodrostat inhibits CYP11B1 and other steroidogenic enzymes in a lesser extent, thereby reducing both cortisol and aldosterone synthesis (Bonnet-Serrano et al., 2022; Varlamov et al., 2021). Compared to metyrapone, osilodrostat has greater potency for CYP11B1 inhibition and a longer half-life. Osilodrostat was approved by both the US FDA and EMA in 2020 for the treatment of Cushing's disease, and in Europe, it is also licensed for use in endogenous Cushing's syndrome (Pivonello et al., 2020). Limited published data on ACC suggest that osilodrostat can effectively control hypercortisolism, improving patients' quality of life (Haissaguerre et al., 2020; Tabarin et al., 2022) . Its rapid action, safety profile, and reduced number of daily doses make it a favorable option. Starting suggested dose is 1-2 mg orally twice a day, but some clinicians recommend starting with a higher dose (≥10 mg/day) in ACC, with close monitoring of serum cortisol (taking in account minor cross-reactivity with 11-deoxycortisol), and block and replace therapy with hydrocortisone as needed (Tabarin et al., 2022). Electrocardiogram changes or testosterone elevation in women are potential side effects that need to be addressed (Fleseriu & Biller, 2022). Further investigation into adrenostatic properties is warranted (Sawabe et al., 2024).

3. Ketoconazole

Ketoconazole, an antifungal agent, also inhibits several steroidogenic enzymes, reducing cortisol synthesis. Ketoconazole should be avoided at the initiation of mitotane therapy because both substances are potentially hepatotoxic, making it difficult to attribute hepatotoxicity to one drug or the other (Fassnacht et al., 2018). However, the addition of ketoconazole to the combination of metyrapone and mitotane can increase overall efficacy (Corcuff et al., 2015). The dosage commonly starts at 200-600 mg/day, with a maintenance dose of 600-800 mg/day administered in 2-3 times, but in cases of severe hypercortisolism, doses up to 1200 mg/day may be required (Castinetti et al., 2014). Elevation of liver enzymes is the most common adverse effect, typically returning to normal within 1-2 weeks after dose reduction or discontinuation. Liver function should be monitored weekly during the first month and monthly thereafter for 6 months. If transaminases exceed 2-3 times the upper limit of normal, alternative therapies may be considered. Other side effects include gastrointestinal symptoms, decreased androgen synthesis resulting in gynecomastia in males, and QT interval prolongation.

4. Etomidate

Etomidate is the only available intravenous medical therapy for hypercortisolism. It has been raditionally used for sedation, has gained attention for its rapid inhibition of cortisol synthesis and is sometimes employed in acute settings to manage severe hypercortisolism. It is a short-acting intravenous agent that inhibits CYP11B1, CYP11A1 (cholesterol side-chain cleavage enzyme), and CYP17A1 (17α-hydroxylase), leading to a reduction in cortisol levels within 12-

24 hours. It has also been shown to inhibit adrenal cortical cell proliferation ACC cultured cell lines. Etomidate doses should be individualized, ideally in an intensive care setting, with frequent assessments of serum cortisol and sedation levels. Clinical guidelines should be consulted for appropriate starting doses and monitoring protocols (Varlamov et al., 2021).

5. Mifepristone and relacorilant

Mifepristone and relacorilant are selective modulators and antagonists of the glucocorticoid receptor employed in the treatment of CS with hypertension and diabetes. Due to their mechanism of action monitoring is based on monitoring of clinical symptoms of CS and the usual hormonal workup is not applicable. This is why even if hypertension is a milestone of CS in ACC experience is reduced in ACC with this drug. A trial involving relacorilant in combination with immunotherapy is in progress (NCT04373265).

References

Annamaria, P., Silvia, P., Bernardo, C., Alessandro, D. L., Antonino, M., Antonio, B., Giuseppe, M., Massimo, R., & Montesani, C. (2015). Adrenocortical carcinoma with inferior vena cava, left renal vein and right atrium tumor thrombus extension. *International Journal of Surgery Case Reports*, *15*, 137–139. https://doi.org/10.1016/J.IJSCR.2015.07.008

Autorino, R., Bove, P., De Sio, M., Miano, R., Micali, S., Cindolo, L., Greco, F., Nicholas, J., Fiori, C., Bianchi, G., Kim, F. J., & Porpiglia, F. (2016). Open Versus Laparoscopic Adrenalectomy for Adrenocortical Carcinoma: A Meta-analysis of Surgical and Oncological Outcomes. *Annals of Surgical Oncology*, *23*(4), 1195–1202. https://doi.org/10.1245/S10434-015-4900-X

Bellantone, R., Lombardi, C. P., & Raffaelli, M. (2015). What is the appropriate role of minimally invasive vs. open surgery for small adrenocortical cancers? *Current Opinion in Oncology*, *27*(1), 44–49. https://doi.org/10.1097/CCO.0000000000000144

Bonnet-Serrano, F., Poirier, J., Vaczlavik, A., Laguillier-Morizot, C., Blanchet, B., Baron, S., Guignat, L., Bessiene, L., Bricaire, L., Groussin, L., Assié, G., Guibourdenche, J., & Bertherat, J. (2022). Differences in the spectrum of steroidogenic enzyme inhibition between Osilodrostat and Metyrapone in ACTH-dependent Cushing syndrome patients. *European Journal of Endocrinology*, *187*(2), 315–322. https://doi.org/10.1530/EJE-22-0208

Castinetti, F., Guignat, L., Giraud, P., Muller, M., Kamenicky, P., Drui, D., Caron, P., Luca, F.,

Donadille, B., Vantyghem, M. C., Bihan, H., Delemer, B., Raverot, G., Motte, E., Philippon, M., Morange, I., Conte-Devolx, B., Quinquis, L., Martinie, M., … Brue, T. (2014). Ketoconazole in cushing's disease: Is it worth a try. *Journal of Clinical Endocrinology and Metabolism, 99*(5), 1623–1630. https://doi.org/10.1210/jc.2013-3628

Claps, M., Cerri, S., Grisanti, S., Lazzari, B., Ferrari, V., Roca, E., Perotti, P., Terzolo, M., Sigala, S., & Berruti, A. (2018). Adding metyrapone to chemotherapy plus mitotane for Cushing's syndrome due to advanced adrenocortical carcinoma. *Endocrine*, *61*(1), 169–172. https://doi.org/10.1007/S12020-017-1428-9

Cooper, A. B., Habra, M. A., Grubbs, E. G., Bednarski, B. K., Ying, A. K., Perrier, N. D., Lee, J. E., & Aloia, T. A. (2013). Does laparoscopic adrenalectomy jeopardize oncologic outcomes for patients with adrenocortical carcinoma? *Surgical Endoscopy*, *27*(11), 4026–4032. https://doi.org/10.1007/S00464-013-3034-0

Corcuff, J. B., Young, J., Masquefa-Giraud, P., Chanson, P., Baudin, E., & Tabarin, A. (2015). Rapid control of severe neoplastic hypercortisolism with metyrapone and ketoconazole. *European Journal of Endocrinology, 172*(4), 473–481. https://doi.org/10.1530/EJE-14-0913

Datta, J., & Roses, R. E. (2016). Surgical Management of Adrenocortical Carcinoma: An Evidence-Based Approach. *Surgical Oncology Clinics of North America*, *25*(1), 153–170. https://doi.org/10.1016/J.SOC.2015.08.011

Dickson, P. V., Kim, L., Yen, T. W. F., Yang, A., Grubbs, E. G., Patel, D., & Solórzano, C. C. (2018). Evaluation, Staging, and Surgical Management for Adrenocortical Carcinoma: An Update from the SSO Endocrine and Head and Neck Disease Site Working Group. *Annals of Surgical Oncology*, *25*(12), 3460–3468. https://doi.org/10.1245/S10434-018-6749-2

Donatini, G., Caiazzo, R., Do Cao, C., Aubert, S., Zerrweck, C., El-Kathib, Z., Gauthier, T., Leteurtre, E., Wemeau, J. L., Vantyghem, M. C., Carnaille, B., & Pattou, F. (2014). Long-term survival after adrenalectomy for stage I/II adrenocortical carcinoma (ACC): a retrospective comparative cohort study of laparoscopic versus open approach. *Annals of Surgical Oncology, 21*(1), 284–291. https://doi.org/10.1245/S10434-013-3164-6

Fassnacht, M., Dekkers, O. M., Else, T., Baudin, E., Berruti, A., De Krijger, R. R., Haak, H. R., Mihai, R., Assie, G., & Terzolo, M. (2018). European society of endocrinology clinical practice guidelines on the management of adrenocortical carcinoma in adults, in collaboration with the European Network for the study of adrenal tumors. In *European Journal of Endocrinology* (Vol. 179, Issue 4, pp. G1–G46). https://doi.org/10.1530/EJE-18-0608

Fleseriu, M., & Biller, B. M. K. (2022). Treatment of Cushing's syndrome with osilodrostat: practical applications of recent studies with case examples. *Pituitary*, *25*(6), 795–809. https://doi.org/10.1007/S11102-022-01268-2

Gaujoux, S., Mihai, R., Carnaille, B., Dousset, B., Fiori, C., Porpiglia, F., Hellman, P., Iacobone, M., Kraimps, J. L., Donatini, G., Langenhuijsen, J., Lorenz, K., Mathonnet, M., Mirallié, E., Blanchard, C., van Dijkum, E. N., Raffaelli, M., Rayes, N., Sébag, F., … Zinzindohoue, F. (2017). *European Society of Endocrine Surgeons (ESES) and European Network for the Study of Adrenal Tumours (ENSAT) recommendations for the surgical management of adrenocortical carcinoma*. *104*(4), 358–376. https://doi.org/10.1002/BJS.10414

Gaujoux, Sébastien, & Brennan, M. F. (2012). Recommendation for standardized surgical management of primary adrenocortical carcinoma. *Surgery*, *152*(1), 123–132. https://doi.org/10.1016/J.SURG.2011.09.030

Gerry, J. M., Tran, T. B., Postlewait, L. M., Maithel, S. K., Prescott, J. D., Wang, T. S., Glenn, J. A., Phay, J. E., Keplinger, K., Fields, R. C., Jin, L. X., Weber, S. M., Salem, A., Sicklick, J. K., Gad, S., Yopp, A. C., Mansour, J. C., Duh, Q. Y., Seiser, N., … Poultsides, G. A. (2016). Lymphadenectomy for Adrenocortical Carcinoma: Is There a Therapeutic Benefit? *Annals of Surgical Oncology*, *23*(Suppl 5), 708–713. https://doi.org/10.1245/S10434-016-5536-1

Haissaguerre, M., Puerto, M., Nunes, M. L., & Tabarin, A. (2020). Efficacy and tolerance of osilodrostat in patients with severe Cushing's syndrome due to non-pituitary cancers. *European Journal of Endocrinology*, *183*(4), L7–L6. https://doi.org/10.1530/EJE-20-0557

Hauch, A., Al-Qurayshi, Z., & Kandil, E. (2015). Factors Associated with Higher Risk of Complications After Adrenal Surgery. *Annals of Surgical Oncology*, *22*(1), 103–110. https://doi.org/10.1245/s10434-014-3750-2

Kenney, L., & Hughes, M. (2023). Adrenocortical Carcinoma: Role of Adjuvant and Neoadjuvant Therapy. *Surgical Oncology Clinics of North America*, *32*(2), 279–287. https://doi.org/10.1016/J.SOC.2022.10.005

Langenhuijsen, J., Birtle, A., Klatte, T., Porpiglia, F., & Timsit, M. O. (2016). Surgical Management of Adrenocortical Carcinoma: Impact of Laparoscopic Approach, Lymphadenectomy, and Surgical Volume on Outcomes-A Systematic Review and Meta-analysis of the Current Literature. *European Urology Focus*, *1*(3), 241–250. https://doi.org/10.1016/J.EUF.2015.12.001

Leboulleux, S., Deandreis, D., Al Ghuzlan, A., Aupérin, A., Goéré, D., Dromain, C., Elias, D., Caillou, B., Travagli, J. P., De Baere, T., Lumbroso, J., Young, J., Schlumberger, M., & Baudin, E. (2010). Adrenocortical carcinoma: is the surgical approach a risk factor of peritoneal carcinomatosis? *European Journal of Endocrinology*, *162*(6), 1147–1153. https://doi.org/10.1530/EJE-09-1096

Lombardi, C. P., Raffaelli, M., Boniardi, M., De Toma, G., Marzano, L. A., Miccoli, P., Minni, F., Morino, M., Pelizzo, M. R., Pietrabissa, A., Renda, A., Valeri, A., De Crea, C., & Bellantone, R. (2012). Adrenocortical carcinoma: effect of hospital volume on patient outcome. *Langenbeck's Archives of Surgery*, *397*(2), 201–207. https://doi.org/10.1007/S00423-011-0866-8

Lombardi, C. P., Raffaelli, M., De Crea, C., Boniardi, M., De Toma, G., Marzano, L. A., Miccoli, P., Minni, F., Morino, M., Pelizzo, M. R., Pietrabissa, A., Renda, A., Valeri, A., & Bellantone, R. (2012). Open versus endoscopic adrenalectomy in the treatment of localized (stage I/II) adrenocortical carcinoma: results of a multiinstitutional Italian survey. *Surgery*, *152*(6), 1158–1164. https://doi.org/10.1016/J.SURG.2012.08.014

Marcadis, A. R., Rubio, G. A., Khan, Z. F., Farra, J. C., & Lew, J. I. (2017). High perioperative morbidity and mortality in patients with malignant nonfunctional adrenal tumors. *The Journal of Surgical Research*, *219*, 259–265. https://doi.org/10.1016/J.JSS.2017.05.116

Maurice, M. J., Bream, M. J., Kim, S. P., & Abouassaly, R. (2017). Surgical quality of minimally invasive adrenalectomy for adrenocortical carcinoma: a contemporary analysis using the National Cancer Database. *BJU International*, *119*(3), 436–443. https://doi.org/10.1111/BJU.13618

Mirallié, E., Blanchard, C., Caillard, C., Rodien, P., Briet, C., Mucci, S., Drui, D., & Hamy, A. (2019). Adrenocortical carcinoma: Impact of surgical treatment. *Annales d'endocrinologie*, *80*(5–6), 308–313. https://doi.org/10.1016/J.ANDO.2019.09.001

Pivonello, R., Fleseriu, M., Newell-Price, J., Bertagna, X., Findling, J., Shimatsu, A., Gu, F., Auchus, R., Leelawattana, R., Lee, E. J., Kim, J. H., Lacroix, A., Laplanche, A., O'Connell, P., Tauchmanova, L., Pedroncelli, A. M., & Biller, B. M. K. (2020). Efficacy and safety of osilodrostat in patients with Cushing's disease (LINC 3): a multicentre phase III study with a double-blind, randomised withdrawal phase. *The Lancet. Diabetes & Endocrinology*, *8*(9), 748–761. https://doi.org/10.1016/S2213-8587(20)30240-0

Reibetanz, J., Rinn, B., Kunz, A. S., Flemming, S., Ronchi, C. L., Kroiss, M., Deutschbein, T., Pulzer, A., Hahner, S., Kocot, A., Germer, C. T., Fassnacht, M., & Jurowich, C. (2019). Patterns of Lymph Node Recurrence in Adrenocortical Carcinoma: Possible Implications

for Primary Surgical Treatment. *Annals of Surgical Oncology*, *26*(2), 531–538. https://doi.org/10.1245/S10434-018-6999-Z

Sawabe, F., Hayafusa, R., Kosugi, R., & Ariyasu, H. (2024). A Case of an Ectopic ACTH-Producing Tumor With Adrenal Shrinkage During Osilodrostat Administration. *JCEM Case Reports*, *2*(2). https://doi.org/10.1210/JCEMCR/LUAE008

Sinclair, T. J., Gillis, A., Alobuia, W. M., Wild, H., & Kebebew, E. (2020). Surgery for adrenocortical carcinoma: When and how? *Best Practice and Research: Clinical Endocrinology and Metabolism*, *34*(xxxx), 101408. https://doi.org/10.1016/j.beem.2020.101408

Tabarin, A., Haissaguerre, M., Lassole, H., Jannin, A., Paepegaey, A. C., Chabre, O., & Young, J. (2022). Efficacy and tolerance of osilodrostat in patients with Cushing's syndrome due to adrenocortical carcinomas. *European Journal of Endocrinology*, *186*(2). https://doi.org/10.1530/EJE-21-1008

Terzolo, M., Fassnacht, M., Perotti, P., Libé, R., Kastelan, D., Lacroix, A., Arlt, W., Haak, H. R., Loli, P., Decoudier, B., Lasolle, H., Quinkler, M., Haissaguerre, M., Chabre, O., Caron, P., Stigliano, A., Giordano, R., Zatelli, M. C., Bancos, I., … Berruti, A. (2023). Adjuvant mitotane versus surveillance in low-grade, localised adrenocortical carcinoma (ADIUVO): an international, multicentre, open-label, randomised, phase 3 trial and observational study. *The Lancet. Diabetes & Endocrinology*, *11*(10), 720–730. https://doi.org/10.1016/S2213-8587(23)00193-6

Turla, A., Laganà, M., Grisanti, S., Abate, A., Ferrari, V. D., Cremaschi, V., Sigala, S., Consoli, F., Cosentini, D., & Berruti, A. (2022). Supportive therapies in patients with advanced adrenocortical carcinoma submitted to standard EDP-M regimen. *Endocrine*, *77*(3), 438–443. https://doi.org/10.1007/S12020-022-03075-Y

Varlamov, E. V., Han, A. J., & Fleseriu, M. (2021). Updates in adrenal steroidogenesis inhibitors for Cushing's syndrome - A practical guide. *Best Practice & Research. Clinical Endocrinology & Metabolism*, *35*(1). https://doi.org/10.1016/J.BEEM.2021.101490

Wu, K., Liu, Z., Liang, J., Tang, Y., Zou, Z., Zhou, C., Zhang, F., & Lu, Y. (2018). Laparoscopic versus open adrenalectomy for localized (stage 1/2) adrenocortical carcinoma: Experience at a single, high-volumecenter. *Surgery*, *164*(6), 1325–1329. https://doi.org/10.1016/J.SURG.2018.07.026
